# Associations of genetically predicted interleukin-6 and tumor necrosis factor signaling pathways with mortality among persons with colorectal cancer: a two-sample Mendelian randomization

**DOI:** 10.1186/s12916-026-04736-9

**Published:** 2026-03-06

**Authors:** Martina Bouka, Katharina Nimptsch, Thu Thi Pham, Emmanouil Bouras, Afroditi Kanellopoulou, Amanda I. Phipps, Bethany Van Guelpen, Hermann Brenner, Li Li, Loïc Le Marchand, Konstantinos K. Tsilidis, Tobias Pischon

**Affiliations:** 1 Molecular Epidemiology Research Group, Max-Delbrück-Center for Molecular Medicine in the Helmholtz Association (MDC), Berlin, Germany; 2 Institute of Public Health, Charité—Universitätsmedizin Berlin, Corporate Member of Freie Universität Berlin and Humboldt-Universität zu Berlin, Berlin, Germany; 3 Clinical Trial Service Unit and Epidemiological Studies Unit, Nuffield Department of Population Health, University of Oxford, Oxford, UK; 4 Department of Epidemiology and Biostatistics, School of Public Health, Imperial College London, London, UK; 5 Department of Hygiene and Epidemiology, University of Ioannina School of Medicine, Ioannina, Greece; 6 Public Health Sciences Division, Fred Hutchinson Cancer Center, Seattle, WA USA; 7 Department of Epidemiology, University of Washington, Seattle, WA USA; 8 Department of Diagnostics and Intervention, Oncology Unit, Umeå University, Umeå, Sweden; 9 Wallenberg Centre for Molecular Medicine, Umeå University, Umeå, Sweden; 10 Division of Clinical Epidemiology and Aging Research, German Cancer Research Center (DKFZ), Heidelberg, Germany; 11 Division of Preventive Oncology, German Cancer Research Center (DKFZ) and National Center for Tumor Diseases (NCT), Heidelberg, Germany; 12 German Cancer Consortium (DKTK), German Cancer Research Center (DKFZ), Heidelberg, Germany; 13 Department of Family Medicine, University of Virginia, Charlottesville, VA USA; 14 University of Hawaii Cancer Center, Honolulu, HI USA; 15 Max-Delbrück-Center for Molecular Medicine in the Helmholtz Association (MDC), Biobank Technology Platform, Berlin, Germany; 16 Charité—Universitätsmedizin Berlin, Corporate Member of Freie Universität Berlin and Humboldt-Universität zu Berlin, Berlin, Germany

**Keywords:** IL-6, TNF-α, Colorectal cancer, Inflammation, Mortality, Mendelian randomization

## Abstract

**Background:**

Despite significant progress in identifying risk factors for colorectal cancer (CRC), factors influencing survival in people with CRC remain less understood. Pro-inflammatory cytokines like interleukin-6 (IL-6) and tumor necrosis factor-alpha (TNF-α) have been implicated in cancer progression and may influence CRC outcomes. We investigated associations between genetically predicted levels of IL-6 and TNF-α signaling pathways and mortality in people with CRC.

**Methods:**

We conducted a two-sample Mendelian randomization (MR) analysis using cis-acting single nucleotide polymorphisms (SNPs) associated with soluble IL-6 receptor alpha (sIL6-RA) and IL-6 signal transducer gp130 (IL6ST), representing IL-6 signaling, and with TNF-α, and its soluble receptors (sTNF-R1, sTNF-R2). SNPs were obtained separately from two large genome-wide association studies (GWAS): deCODE and UK Biobank (UKB). The outcome was CRC-specific mortality among 16,964 CRC cases (4010 deaths) in the Genetics and Epidemiology of Colorectal Cancer Consortium (GECCO). Analyses were stratified by tumor site and stage. The inverse variance weighted (IVW) method, incorporating a correlation matrix for dependent SNPs, was used for primary analyses. Because literature links TNF-α to CRC incidence, we additionally performed a simulation study to evaluate the potential impact of collider bias resulting from restricting analyses to CRC cases.

**Results:**

Genetically predicted sIL6-RA was weakly positively associated with CRC-specific mortality (deCODE-SNPs (*n* = 13) HR per 1 SD increase: 1.06; 95% CI: 1.00–1.12; UKB-SNPs (*n* = 11) HR: 1.09; 95% CI: 1.02–1.17). Genetically proxied IL6ST levels showed no association with CRC-specific mortality in the overall sample (deCODE-SNPs (*n* = 19) HR: 1.04; 95% CI: 0.90–1.21; UKB-SNPs (*n* = 9) HR: 1.11; 95% CI: 0.87–2.42), while higher IL6ST levels were associated with increased mortality among patients with stage 2/3 disease (deCODE-SNPs (*n* = 19) HR: 1.45; 95% CI: 1.10–1.91; UKB-SNPs (*n* = 9) HR: 1.87; 95% CI: 1.22–2.89). No associations were observed for TNF-α, sTNF-R1, or sTNF-R2. Findings for all exposures were consistent across both GWAS datasets. Simulation analyses for TNF-α indicated collider bias was present but limited in magnitude.

**Conclusions:**

Our findings suggest that IL-6 signaling may play a role in CRC progression although of limited magnitude, whereas TNF-related pathways appear less relevant for prognosis.

**Supplementary Information:**

The online version contains supplementary material available at 10.1186/s12916-026-04736-9.

## Background

Colorectal cancer (CRC) is the third most common in terms of incidence and the second leading cause of cancer-related deaths worldwide [1]. Significant progress has been made in understanding the risk factors for CRC development, such as diet, nutrition, and lifestyle factors, including obesity [2–4]. Thus, the most recent report of the World Cancer Research Fund (WCRF) concluded that there is strong evidence that being overweight or obese is associated with a higher CRC risk [5, 6], which is supported by large Mendelian randomization (MR) studies [7–11]. However, less is known about factors that influence prognosis and mortality in people with CRC.

Emerging evidence suggests that adiposity may play a role also for mortality among persons with CRC [12]. Most studies to date have assessed pre-diagnostic BMI, which helps avoid bias from weight loss due to disease or treatment. Cohort studies and meta-analyses [13–15] as well as a recent MR study [16] suggest that higher BMI before diagnosis is associated with increased CRC-specific and all-cause mortality while studies on post-diagnostic BMI are fewer and more prone to reverse causation. Nevertheless, recent evidence from the WCRF Global Cancer Update Programme’s systematic review and meta-analysis also highlights the potential prognostic value of post-diagnostic adiposity [17]. While these findings suggest that adiposity may influence CRC outcomes, the underlying biological mechanisms remain unclear. Chronic low-grade inflammation is one plausible pathway, as adipose tissue acts as an endocrine organ secreting numerous cytokines and chemokines involved in inflammation and cancer progression [18, 19].

Several cytokines, including interleukin-6 (IL-6) and tumor necrosis factor-alpha (TNF-α), are upregulated in CRC and are associated with poor prognosis in patients [20]. Both are produced by a variety of cells including adipose tissue–resident macrophages as well as cells in other tissues, and their blood concentrations are positively correlated with adiposity measures [21–24].

In addition, the soluble IL-6 receptor (sIL6-RA) circulates in blood, serving as binding reservoir but also modulating IL-6–mediated signaling. sIL6-RA also enables trans-signaling, allowing IL-6 to act on cells that do not express the membrane-bound receptor, thereby broadening its pro-inflammatory effects. Thus, when IL-6 binds to either the membrane-bound IL6-RA or to sIL6-RA, the complex may associate with the signal transducer gp130 (IL6ST), thereby initiating downstream activation of the JAK/STAT pathway and promoting inflammatory responses [25, 26]. Soluble receptors of TNF-α (sTNF-R1, sTNF-R2) primarily function to bind and regulate circulating TNF-α, acting as reservoirs that can either stabilize or attenuate TNF-α activity depending on the physiological context [27, 28].

These cytokines play a crucial role in the inflammatory response, including hepatic production of acute-phase proteins such as C-reactive protein (CRP) [24]. Several observational studies have reported positive associations between post-diagnostic and preoperative CRP concentrations and CRC prognosis [29, 30]. In contrast, results from Mendelian randomization (MR) studies have been inconsistent, with most studies showing no overall association of genetically predicted CRP concentrations on mortality in persons with CRC, although some found significant associations for individual SNPs [31–33].

While CRP has been widely studied, with no evidence for a causal role in CRC-specific mortality, here we investigated the inflammatory signatures of IL-6 and TNF-α in relation to mortality among individuals with CRC. Observational studies have reported elevated IL-6 and TNF-α levels in individuals with CRC compared to healthy controls [34–36], with higher IL-6 levels associated with more advanced disease, metastasis, and poorer survival [36–39]. Some studies have also linked higher TNF-α levels to shorter overall survival, although findings are mixed [34, 40, 41]. For the soluble receptors or IL6ST, evidence is limited. Understanding the potential roles of these cytokines is important, as it may provide insights into biological mechanisms underlying CRC progression and identify potential targets for improving prognosis and personalized treatment. Therefore, we explored whether the inflammatory signature defined by IL-6 and TNF-α is associated with CRC-specific mortality in people with CRC using an MR approach.

## Methods

We conducted a two-sample MR analysis to evaluate the potential relationship between genetically predicted levels of IL-6, sIL6-RA, IL6ST, TNF-α, sTNF-R1, and sTNF-R2 and CRC-specific mortality. MR is a technique that uses genetic variation as a tool to investigate genetic causal relationships between modifiable exposures and health outcomes [42]. It is based on the principle that genetic variants are randomly assigned at conception, which makes them less susceptible to confounding factors that often affect observational studies [43]. MR also helps to mitigate the issue of reverse causation, where disease progression may influence the exposure of interest—a particular concern in studies of survival [42]. In all MR analysis, the selected genetic instruments have to meet the following criteria: (a) be strongly associated with the circulating levels of the cytokine (relevance assumption), (b) be independent of any potential confounder of the cytokine-CRC mortality association (independence assumption), and (c) affect CRC mortality only through the cytokine being instrumented and not via any other biological pathway (exclusion restriction assumption) [44]. To measure the overall strength of the genetic instruments for each exposure, the *F*-statistic was calculated using the formula ((*n* − *k* − 1)/*k*)/((1 − *R*^2^)/*R*^2^), where *R*^2^ is the total proportion of the explained variance of the cytokine by the selected genetic instruments, *k* is the number of selected instruments, and *n* is the GWAS sample size of the SNP-cytokine association [45].

Summary statistics for genetic associations with cytokine levels (exposures) and CRC-specific mortality (outcome) were obtained from separate, non-overlapping genome-wide association study (GWAS) datasets.

### Exposure data

Genetic instruments for circulating cytokines and soluble receptors were derived separately from two recent large protein quantitative trait loci (pQTL) GWAS. The first by Ferkingstad et al. was conducted in 35,559 Icelanders within the deCODE study, where plasma levels of 4719 proteins were measured using the SomaScan v4 platform and tested against 27.2 million sequence variants imputed from whole-genome sequencing of 49,708 individuals [46]. Association analyses were adjusted for age, sex, and sample age (time since blood collection). The second by Sun et al. was based on 46,861 participants in the UK Biobank (UKBB), with plasma protein levels measured using the Olink Explore 1536 platform and analyzed against genome-wide genetic variation [47]. Association analyses adjusted for age, age^2^, sex, sex × age, age^2^ × sex, batch, UKBB center, UKBB genetic array, time between blood sampling and measurement, and the first 20 genetic principal components. Both GWAS were included to make use of independent large-scale datasets and ensure that the most well-powered instruments could be selected for each biomarker, and to assess whether results were consistent across studies.

From both GWAS, we extracted summary statistics for cis-acting variants (± 500 kb of the gene encoding each biomarker). We first considered variants reaching conventional genome-wide significance (*P* < 5 × 10^−8^). No suitable cis SNPs were identified for IL-6 in either GWAS. For sIL6-RA, the resulting total *R*^2^ of SNPs was sufficient to apply conventional LD clumping at *r*^2^ < 0.001. For other biomarkers, the LD threshold was relaxed to *r*^2^ < 0.1 to increase instrument numbers and improve statistical power. Instrument sets with total variance explained *R*^2^ < 1% were excluded. We also explored a more lenient threshold (*P* < 1 × 10^−6^), but this did not identify variants for IL-6 either and only modestly increased instrument numbers for other biomarkers, without substantially improving power.

**Fig. 1:**
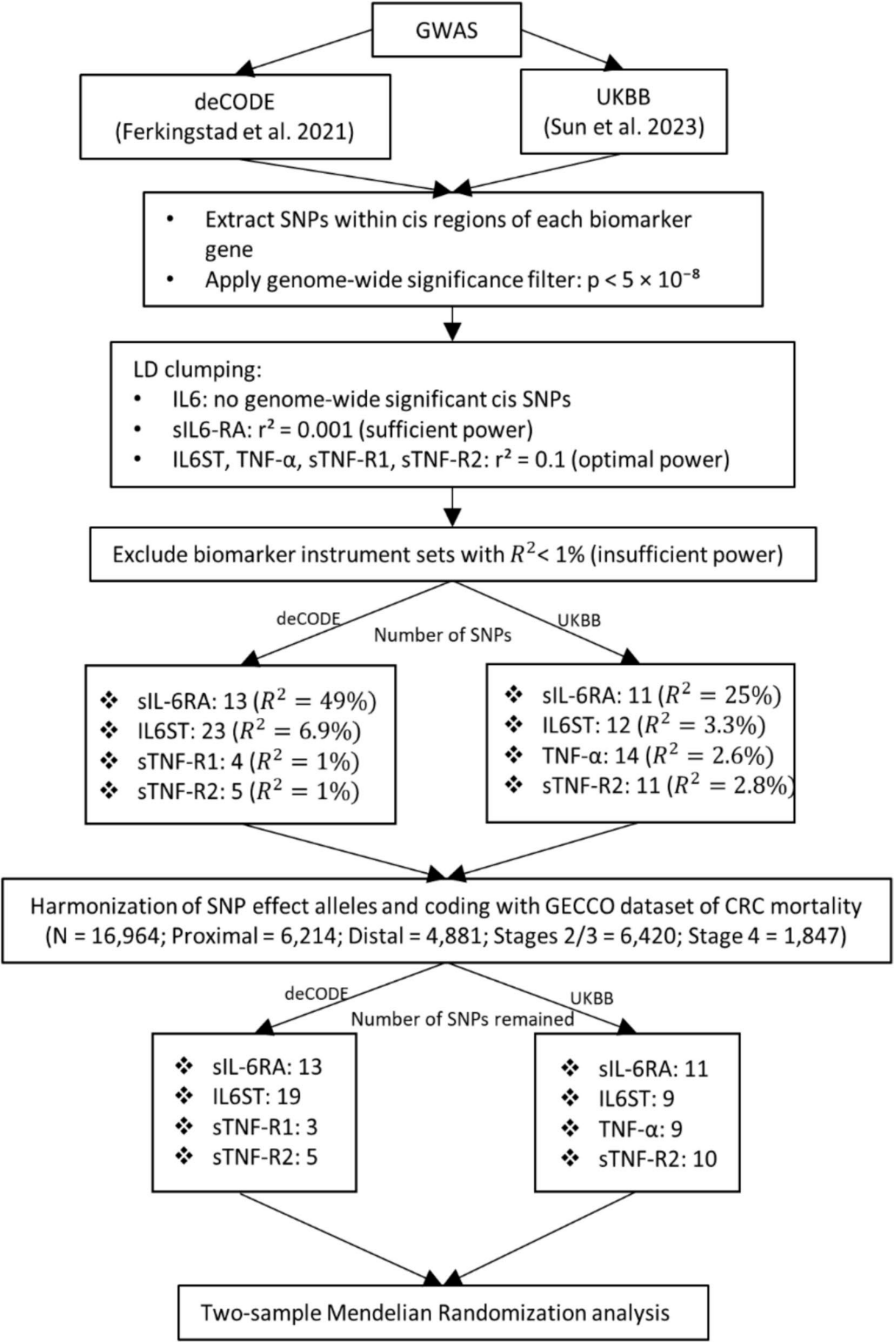
Instrument selection for two-sample Mendelian randomization

Following these criteria, the deCODE GWAS provided instruments for sIL6-RA (13 SNPs, *R*^2^ = 49%), IL6ST (23 SNPs, *R*^2^ = 6.9%), sTNF-R1 (4 SNPs, *R*^2^ = 1.0%), and sTNF-R2 (5 SNPs, *R*^2^ = 1.0%). The UKBB GWAS provided instruments for sIL6-RA (11 SNPs, *R*^2^ = 25%), IL6ST (12 SNPs, *R*^2^ = 3.3%), TNF-α (14 SNPs, *R*^2^ = 2.6%), and sTNF-R2 (11 SNPs, *R*^2^ = 2.8%). For all biomarkers, the *F*-statistics were above 10. Characteristics of all SNP instruments, including effect estimates and strength metrics, are shown in Additional file 1: Table 1, and the SNP selection workflow is illustrated in Fig. 1.

### Outcome data

Genetic association estimates (EAF, beta coefficients, SEs, *P* values, and sample sizes) for colorectal cancer (CRC)-specific mortality were obtained from the Genetics and Epidemiology of Colorectal Cancer Consortium (GECCO) and the Colon Cancer Family Registry (CCFR) [48]. These consortia had combined data from 15 cohort and clinical studies, including 16,964 individuals of European ancestry diagnosed with incident, invasive CRC. During a median follow-up of 13.8 years, 4010 CRC-related deaths had been recorded. Genotyping had been conducted across multiple platforms, followed by standard quality control procedures, and imputation had been performed using the Haplotype Reference Consortium reference panel (release 1.1). All analyses were performed by the consortia prior to data provision. Specifically, Cox proportional hazards regression models were used to assess associations between individual SNPs and CRC-specific mortality under an additive genetic model. Models were adjusted for age at diagnosis, sex, study-specific factors, genotyping platform, and the first five principal components to account for population structure. In addition to overall analyses, stratified analyses were conducted by anatomical tumor site: proximal colon (6214 cases, 1433 deaths), distal colon (4881 cases, 978 deaths), rectum (4749 cases, 1045 deaths), and by tumor stage: stage I (3338 cases, 157 deaths), stages II–III (6420 cases, 1209 deaths), stage IV (1847 cases, 1448 deaths). Further details on cohort characteristics, genotyping, quality control, and imputation procedures are available in the original GWAS publication [49].

Analyses for stage-specific outcomes in our study excluded stage I cases in the primary analysis due to the very limited number of CRC-related deaths in this group, which, together with the small number of available genetic instruments for our biomarkers, will result in severely limited statistical power and unreliable MR estimates. Stage I–specific MR analyses were instead conducted as an exploratory analysis.

CRC mortality estimates for each selected biomarker-associated SNP are presented in Additional file 1: Table 2.

### Statistical analysis

#### Mendelian randomization analysis

Two-sample MR using summary association data was performed for CRC-specific mortality overall, as well as stratified by anatomical tumor subsite and stage of cancer (Additional file 2: Fig. 1) [46, 47]. For inflammatory biomarkers with instruments available from both GWAS (deCODE and UK Biobank), analyses were conducted separately for each dataset. To account for dependency among SNPs, a correlation matrix of the genetic instruments was incorporated for all biomarkers except sIL6-RA. MR estimates were obtained using the random-effects inverse variance weighted (IVW) method, and associations are reported per one standard deviation (SD) increase in biomarker levels, where SD refers to the study-specific standardized exposure (UKBB: inverse-rank–normalized Olink NPX; deCODE: standardized log-RFU SomaScan measures). To account for multiple testing across the four exposures available in each GWAS, we applied Bonferroni correction (corrected *α* = 0.0125).

#### Calculations of the minimally detectable effect (hazard ratio)

We calculated the minimally detectable hazard ratio (HR) for CRC-specific mortality as a binary outcome using the method described by Burgess [45] with a type 1 error *α* = 0.05. This method does not take the time until the end of follow-up into account. Based on the number of total CRC cases and deaths in the outcome dataset, our study had at least 80% power to detect the following HRs per SD: in deCODE, 1.05 for sIL6-RA, 1.14 for IL6ST, and 1.42 for both sTNF-R1 and sTNF-R2; in UK Biobank, 1.07 for sIL6-RA, 1.21 for IL6ST, 1.24 for TNF-α, and 1.23 for sTNF-R2 (Additional file 3: Table 1) [14, 46, 47, 50–55].

#### Sensitivity analyses

To examine potential violation of the second and third MR assumptions, the Cochran’s *Q* statistic was calculated which assesses the degree to which variation in the individual SNP-specific effect estimates reflects true heterogeneity between genetic variants rather than random variation due to sampling error [56]. MR-Egger regression was performed to detect potential directional pleiotropy, with statistical evidence assessed by the intercept term being different from zero. In addition, leave-one-out (LOO) analyses were conducted by iteratively excluding each genetic variant to assess the influence of individual SNPs on the overall estimate. When a correlation matrix was included to account for linkage disequilibrium between genetic instruments, statistical sensitivity methods such as weighted median and MR-PRESSO (which detects and corrects for potential outlier variants that may bias the associations) could not be performed, as these methods require independent instruments. Therefore, they were implemented only for sIL6-RA. We also performed colocalization analyses, where relevant, to assess whether genetic variants associated with inflammatory protein levels also influenced CRC-specific mortality through shared causal variants. Finally, to further assess potential off-target pathways, we queried all genetic instruments in the GWAS Catalog [57] to identify previously reported genome-wide significant trait associations.

Analyses were implemented in the statistical software R (version 4.4.3) using the TwoSampleMR, MendelianRandomization, MRPRESO, and coloc packages. This study adheres to the guidelines for strengthening the reporting of Mendelian randomization studies STROBE-MR (Additional file 4: STROBE-MR checklist) [58, 59].

#### Collider bias simulation

Collider bias occurs when an exposure and outcome (or factors causing these) each influence a common third variable and that variable or collider is controlled for by design or analysis [60]. In MR studies of CRC survival, collider bias can arise because analyses are restricted to individuals with CRC, and certain exposures are also associated with CRC incidence. This may induce spurious associations between the previously independent genetic variants and measured or unmeasured confounders, violating the second MR assumption (independence) [51]. In our case, there is evidence that TNF-α is associated with CRC risk [55, 61], raising concern about potential collider bias influencing our results.

Some statistical approaches have been proposed to correct for collider bias in MR studies including the methods by Dudbridge et al. [62], the Slope-Hunter method [63], and the Corrected Weighted Least Squares (CWLS) method by Cai et al. [64]. All three approaches estimate a bias-correcting factor by regressing SNP–survival associations on SNP–incidence associations and adjusting the survival effect estimates accordingly. The Dudbridge method assumes no genetic correlation between incidence and survival. Slope-Hunter attempts to overcome this limitation by clustering variants into those that influence incidence only and those with pleiotropic effects. The CWLS method also relies on the assumption of no genetic correlation but aims to improve statistical performance through an intercept-free regression model. For a detailed overview of each method and their underlying assumptions, we refer readers to Mitchell et al. [51]. Among other limitations, these methods are designed to correct for collider bias but require a large number of SNPs. Given the limited number of instruments available for TNF-α, their application would not yield valid results in our study. Instead, we drew on ideas described in Mitchell et al. and previously implemented by Noyce et al. [52] to perform a simulation study. The aim was not to correct the estimates, but to assess whether collider bias was present in our data and, if so, to approximate its magnitude.

The rationale was as follows: starting from the assumption that TNF-α has no effect on CRC mortality, we simulated data to assess whether collider bias alone could generate spurious associations. In these simulations, SNPs were set to influence TNF-α levels; CRC incidence was modeled as a function of genetically predicted TNF-α, BMI (as a confounder), and age; and CRC mortality was modeled as a function of BMI and age. Age was included to capture its established role in both CRC incidence and survival, providing a realistic time-related component to the simulation. We then examined whether an apparent association between genetically predicted TNF-α and CRC survival emerged, and if so, whether its magnitude could explain our empirical results; and whether TNF-associated SNPs became spuriously associated with BMI among CRC cases, thus violating the independence assumption of MR.

The simulation proceeded in seven steps:

1. A large synthetic population (*N* = 500,000) was generated in which each individual carried alleles at two independent TNF-associated SNPs (*r*^2^ = 0.001).
2. BMI and age values were generated to approximate reported distributions in the GECCO studies, using pre-diagnostic BMI data from Campbell et al. [50] and baseline age distributions from Wei et al. [54].
3. CRC incidence was simulated using a Cox model as a function of BMI, age, and TNF-α.
4. CRC mortality status was simulated using an exponential proportional hazards model as a function of BMI and age.
5. Only CRC cases were retained for analysis.
6. MR analyses were performed to test for associations between genetically predicted TNF-α and CRC mortality, as well as between TNF-α SNPs and BMI.
7. The simulation was repeated 1000 times to obtain the distribution of MR estimates expected under collider bias alone.

In contrast, we did not apply the simulation for IL-6, sIL6-RA, or IL6ST, as there is currently no strong evidence linking genetically predicted IL-6 [65, 66], sIL6-RA, or IL6ST levels to CRC incidence. Similarly, we found no published evidence suggesting associations between sTNF-R1 or sTNF-R2 levels and CRC risk.

## Results

After harmonization with the outcome dataset, all selected SNPs were retained for sIL6-RA and sTNF-R2 from the deCODE GWAS, while 19 SNPs remained for IL6ST and 3 SNPs for sTNF-R1. For the UKBB GWAS, all SNPs were retained for sIL6-RA, and 9 SNPs each for IL6ST and TNF-α, as well as 10 SNPs for sTNF-R2.

### IL-6 signaling

For sIL6-RA, the IVW analysis indicated higher CRC-specific mortality in both GWAS (HR per 1 SD increase: 1.06; 95% CI: 1.00–1.12 using deCODE-SNPs and HR: 1.09; 95% CI: 1.02–1.17 using UKBB-SNPs) (Table 1). The association using UKBB-SNPs remained significant (raw *P* = 0.012, adjusted *P* = 0.047) after Bonferroni correction, whereas the one based on deCODE-SNPs (raw* P* = 0.042, adjusted *P* = 0.167) did not. Subsite- and stage-specific analyses were broadly consistent across GWAS, with no further associations observed. Exploratory analyses among stage I patients suggested an association between genetically proxied sIL6-RA and CRC-specific mortality, although these results were based on few events and should be interpreted with caution.

**Table 1:** MR estimates for genetically predicted inflammatory biomarkers on colorectal cancer-specific mortality

Biomarker	deCODE			UKBB		
	IVW HR^a,b,c^ (95% CI)	MR-Egger HR (95% CI)	nSNP^d^	IVW HR (95% CI)	MR-Egger HR (95% CI)	nSNP
sIL-6RA						
Overall	1.06 (1.00, 1.12)	1.09 (0.97, 1.22)	13	1.09 (1.02, 1.17)	1.05 (0.87, 1.26)	11
Proximal colon	1.05 (0.96, 1.15)	0.95 (0.79, 1.15)	13	1.06 (0.96, 1.18)	1.00 (0.77, 1.31)	11
Distal colon	1.08 (0.94, 1.23)	1.30 (0.99, 1.70)	13	1.11 (0.98, 1.26)	1.02 (0.73, 1.42)	11
Rectal	0.98 (0.87, 1.10)	1.03 (0.80, 1.33)	13	1.08 (0.96, 1.22)	1.12 (0.82, 1.53)	11
Stage 1^f^	1.45 (1.09, 1.92)	NA	13	1.45 (1.06, 1.99)	NA	11
Stages 2/3	0.99 (0.90, 1.09)	0.98 (0.80, 1.20)	13	1.04 (0.92, 1.17)	0.80 (0.60, 1.07)	11
Stage 4	1.10 (0.99, 1.22)	1.18 (0.94, 1.48)	13	1.06 (0.95, 1.19)	1.17 (0.87, 1.58)	11
IL6ST						
Overall	1.04 (0.90, 1.21)	1.09 (0.91, 1.30)	19	1.11 (0.87, 1.42)	1.13 (0.84, 1.54)	9
Proximal colon	1.09 (0.85, 1.40)	1.07 (0.79, 1.45)	19	0.98 (0.65, 1.48)	1.12 (0.67, 1.88)	9
Distal colon	1.14 (0.85, 1.54)	1.39 (0.98, 1.98)	19	1.47 (0.88, 2.46)	1.47 (0.75, 2.89)	9
Rectal	1.12 (0.82, 1.52)	1.10 (0.76, 1.59)	19	1.25 (0.77, 2.02)	1.22 (0.68, 2.21)	9
Stage 1	1.05 (0.47, 2.34)	NA	19	0.74 (0.20, 2.67)	NA	
Stages 2/3	1.45 (1.10, 1.91)	1.41 (1.01, 1.97)	19	1.87 (1.22, 2.89)	1.85 (1.09, 3.14)	9
Stage 4	1.21 (0.94, 1.56)	1.11 (0.82, 1.51)	19	1.39 (0.91, 2.11)	1.02 (0.60, 1.71)	9
TNF-α						
Overall	NA	NA	NA	1.19 (0.79, 1.77)	1.34 (0.64, 2.82)	9
Proximal colon	NA	NA	NA	1.50 (0.80, 2.81)	1.94 (0.62, 6.06)	9
Distal colon	NA	NA	NA	0.65 (0.14, 2.93)	0.51 (0.03, 8.24)	9
Rectal	NA	NA	NA	1.09 (0.51, 2.30)	1.52 (0.39, 5.82)	9
Stage 1	NA	NA	NA	1.53 (0.09, *)^e^	NA	3
Stages 2/3	NA	NA	NA	1.60 (0.58, 4.41)	1.83 (0.11, *)	3
Stage 4	NA	NA	NA	1.69 (0.71, 3.99)	4.96 (0.18, *)	3
sTNF-R1						
Overall	0.98 (0.70, 1.36)	0.95 (0.61, 1.49)	3	NA	NA	NA
Proximal colon	1.43 (0.80, 2.55)	1.17 (0.53, 2.58)	3	NA	NA	NA
Distal colon	0.71 (0.36, 1.41)	0.78 (0.31, 1.96)	3	NA	NA	NA
Rectal	0.83 (0.44, 1.58)	0.67 (0.29, 1.56)	3	NA	NA	NA
Stage 1	0.34 (0.07, 1.76)	NA	3	NA	NA	NA
Stages 2/3	1.11 (0.59, 2.07)	0.82 (0.28, 2.34)	3	NA	NA	NA
Stage 4	0.97 (0.56, 1.69)	1.07 (0.47, 2.41)	3	NA	NA	NA
sTNF-R2						
Overall	0.95 (0.67, 1.33)	1.02 (0.63, 1.63)	5	0.95 (0.73, 1.24)	1.10 (0.80, 1.51)	10
Proximal colon	0.60 (0.32, 1.13)	0.49 (0.19, 1.30)	5	0.70 (0.43, 1.14)	0.74 (0.40, 1.36)	10
Distal colon	1.01 (0.33, 3.11)	2.41 (0.75, 7.69)	5	1.10 (0.60, 2.02)	1.59 (0.81, 3.12)	10
Rectal	1.19 (0.60, 2.35)	0.70 (0.27, 1.80)	5	0.97 (0.51, 1.83)	1.18 (0.54, 2.55)	10
Stage 1	0.52 (0.09, 2.84)	NA	5	0.48 (0.09, 2.65)	NA	10
Stages 2/3	0.79 (0.42, 1.50)	0.97 (0.40, 2.36)	5	1.03 (0.63, 1.69)	0.99 (0.55, 1.79)	10
Stage 4	0.84 (0.47, 1.50)	1.01 (0.45, 2.31)	5	0.89 (0.56, 1.39)	1.00 (0.58, 1.73)	10

^a^Hazard ratios (HRs) and 95% confidence intervals (CIs) are presented for the estimated effects of genetically predicted inflammatory biomarkers on colorectal cancer-specific mortality, using Mendelian randomization (MR) with the inverse variance weighted (IVW) and MR-Egger methods

^b^Genetic cis instruments for biomarker levels were derived from two genome-wide association studies (GWAS): deCODE genetics (Ferkingstad et al., 2021) and UK Biobank (UKBB) (Sun et al., 2023)

^c^HRs represent the effect per one standard deviation (SD) increase in biomarker levels

^d^Abbreviations: *nSNP*, number of SNPs; *NA*, not applicable

^e^Upper CIs exceeding 10 are symbolized with *

^f^Stage 1–specific MR estimates are considered as part of exploratory analysis because of insufficient statistical power (157 deaths among 3338 stage 1 cases) and thus MR-Egger (or other) sensitivity analysis was not performed

For IL6ST, the IVW analysis suggested no association with CRC-specific mortality overall (HR: 1.04; 95% CI: 0.90–1.21 using deCODE-SNPs and HR: 1.11; 95% CI: 0.87–1.42 using UKBB-SNPs) (Table 1). In stage-stratified analyses, positive associations were observed for stages 2/3 disease (HR: 1.45; 95% CI: 1.10–1.91; raw *P* = 0.009, adjusted *P* = 0.036 using deCODE-SNPs and HR: 1.87; 95% CI: 1.22–2.89; raw *P* = 0.004, adjusted *P* = 0.018 using UKBB-SNPs) and both remained significant after Bonferroni correction. Subsite-specific analyses did not indicate other associations.

Across both biomarkers, MR-Egger regression provided effect estimates of similar direction and magnitude (Table 1). Weighted median analyses for sIL6-RA produced results consistent with the IVW estimates, and MR-PRESSO did not identify outlier SNPs. No evidence of substantial heterogeneity was indicated with non-significant Cochran’s *Q* statistics (all *P* > 0.05). Evidence for horizontal pleiotropy was observed only in the IL6ST distal colon analysis using deCODE-SNPs and stage 4 disease using UKBB-SNPs (MR-Egger intercept *P* = 0.04), though these *P* values were marginal and since MR-Egger estimates were highly consistent with the IVW results, pleiotropy is unlikely to materially affect the conclusions. LOO analyses for sIL6-RA and IL6ST indicated that results were generally robust. Minor shifts in effect estimates were observed when excluding individual SNPs in some subsite-specific analyses, but overall patterns remained consistent using either deCODE or UKBB SNPs. Finally, GWAS Catalog annotation indicated that IL6RA instruments were largely specific to IL-6 pathway traits, with no meaningful off-target associations. For IL6ST, some instruments showed prior associations with anthropometric traits (height, lean body mass) and immune-mediated diseases. However, these SNPs were present only in one of the two exposure GWAS (deCODE), and MR estimates were consistent across both GWAS sources, including the dataset in which no such off-target associations were observed. Together with the pleiotropy-robust and leave-one-out sensitivity analyses, these observations suggest that the IL6ST findings are unlikely to be materially influenced by horizontal pleiotropy.

Given the observed associations for sIL6-RA and IL6ST, we conducted colocalization analyses in the overall CRC sample to examine whether genetic variants at these loci shared causal signals with CRC-specific mortality. Analyses were performed separately using instruments from the deCODE and UKBB GWAS, and results were highly consistent across datasets. For sIL6-RA, the posterior probability for a shared causal variant was low (PP.H4 = 4.6% using deCODE SNPs; 4.5% using UKBB SNPs), with most of the probability supporting an association confined to the IL6R locus (PP.H1 = 92.5% using deCODE SNPs; 92.6% using UKBB SNPs). Similarly, for IL6ST, PP.H4 values were low (PP.H4 = 2.3% using deCODE-SNPs; 2.2% using UKBB-SNPs), with most of the probability again supporting locus-specific associations (PP.H1 = 93.4% using deCODE-SNPs; 93.2% using UKBB-SNPs). These results suggest that IL-6 signaling variants do not share causal signals with CRC-specific mortality.

Forest plots for the MR and LOO analyses of sIL6-RA and IL6ST are provided in Additional file 2: Figs. 2–7. Results from sensitivity analyses for these markers (weighted median, MR-PRESSO, heterogeneity and pleiotropy tests, colocalization) are presented in Additional file 3: Tables 2–5.

### TNF-α signaling

For genetically predicted TNF-α, the IVW analysis using UKBB-SNPs suggested no association with CRC-specific mortality (HR: 1.19; 95% CI: 0.79–1.77). Similarly, for the soluble receptors, sTNF-R1 (HR: 0.98; 95% CI: 0.70–1.36 using deCODE SNPs) and sTNF-R2 (HR: 0.95; 95% CI: 0.67–1.33 using deCODE-SNPS and HR: 1.02; 95% CI: 0.63–1.63 using UKBB-SNPs), no associations were observed with overall CRC-specific mortality. Subsite- and stage-stratified analyses did not reveal any further associations (Table 1).

For TNF-α and its soluble receptors, MR-Egger regression provided effect estimates consistent with the IVW results, though with wider confidence intervals (Table 1). No evidence of substantial heterogeneity was indicated, except in analyses of TNF-α for distal colon cancer and sTNF-R2 for distal colon cancer using deCODE SNPs (Cochran’s *P* = 0.04 for both). A weak indication of pleiotropy was observed only for sTNF-R2 in the distal colon analysis using deCODE-SNPs (MR-Egger intercept *P* = 0.04), though the MR-Egger estimates were consistent with the IVW results. LOO analyses did not suggest that results were driven by any single SNP for TNF-α, sTNF-R1, or sTNF-R2. Finally, the GWAS Catalog annotation did not reveal any off-target pathways relevant to CRC prognosis.

Forest plots for the MR and LOO analyses of TNF-α and its receptors are provided in Additional file 2: Figs. 8–14. Results from heterogeneity and pleiotropy tests for these markers are presented in Additional file 3: Table 4.

### Collider bias

We performed simulations under the assumption of no effect of TNF-α on CRC-specific mortality. In this setting, any associations observed would necessarily arise from collider bias. Full details on simulation parameterization are provided in Additional file 2: Collider bias simulation.

On average, the MR estimator produced a very small upward bias (mean *β* = 0.022, HR ≈ 1.02). Across 1000 simulations, the distribution of effect estimates varied around the null, with hazard ratios ranging roughly between 0.81 and 1.31. This indicates that while individual simulated datasets can yield estimates away from the null, the average result remained close to no effect. The performance metrics confirmed this pattern: bias was small (0.022), type I error was slightly inflated (6.1% vs expected 5%), and coverage of the 95% confidence interval was slightly below nominal (93.9%). To directly assess collider bias, we examined associations between TNF-α SNPs and BMI among CRC cases. On average, 0.14 SNPs per run showed a spurious association, and in ~ 13% of simulations at least one SNP was significant (maximum observed per run = 2 SNPs). This indicates that collider bias was present but modest, and unlikely to explain large effect estimates. Taken together, the simulations suggest that in the absence of an effect of TNF-α on CRC-specific mortality, MR estimates remain close to no effect, with only small bias and occasional spurious SNP–BMI associations due to collider bias (Additional file 2: Figs. 15–17 and Additional file 3: Table 6).

## Discussion

In this two-sample MR study, we investigated the role of genetically predicted IL-6 signaling (proxied by sIL6-RA and IL6ST), TNF-α, and soluble TNF receptors (sTNF-R1 and sTNF-R2) in colorectal cancer (CRC)-specific mortality among individuals with CRC. We found modest evidence for IL-6 signaling in relation to CRC-specific mortality. Genetically predicted higher sIL6-RA levels were consistently associated with slightly increased mortality across both GWAS, with the association using UKBB-SNPs surviving the Bonferroni correction. IL6ST showed associations only in stage-stratified analyses, with higher genetically predicted levels linked to increased mortality in stage 2/3 disease. In contrast, we found no consistent evidence that TNF-α or its soluble receptors (sTNF-R1 and sTNF-R2) were associated with CRC-specific mortality. Interpreting these findings in the context of statistical power, the observed sIL6-RA effect estimates (HRs ~ 1.06–1.09 per SD) were above the minimally detectable hazard ratios of our study, indicating that these associations fall within the range our analyses were powered to detect, although their proximity to this boundary suggests that any effect of IL-6 signaling on CRC-specific mortality is likely modest. In contrast, the overall IL6ST estimates were smaller in magnitude (HRs ~ 1.04–1.11) and lay below or close to the minimally detectable HRs in both deCODE (1.14) and UKBB (1.21), suggesting limited power to detect effects of this size in the overall CRC population. For TNF-α and the soluble TNF receptors, our study was powered primarily to detect relatively large effects, and the absence of clear associations therefore does not entirely rule out the possibility of small effects. Sensitivity analyses, including MR-Egger, weighted median, MR-PRESSO, heterogeneity and pleiotropy testing, and LOO analyses, indicated that the results were generally robust, with no evidence of directional pleiotropy and no indication that findings were materially driven by single SNPs. Collider bias simulations further suggested that any bias introduced by restricting analyses to CRC cases was modest and unlikely to generate spurious associations. Overall, our findings provide some evidence for a role of IL-6 signaling in CRC-specific mortality, although of limited magnitude, while not supporting a major contribution of TNF-α pathways.

Evidence from observational studies suggests that elevated IL-6 levels may be associated with poorer survival in individuals with CRC. Several case–control studies have reported higher circulating post-diagnostic IL-6 levels in individuals with CRC compared to healthy controls [34–36]. In addition, some of these same studies [35, 36], along with others [37, 38], have shown that higher IL-6 levels are related to more advanced disease, metastasis, and shorter survival in CRC patients. A systematic review and meta-analysis also confirmed that serum IL-6 expression was highly correlated with poor 5-year overall survival [39]. Another study based on post-treatment plasma samples from CRC patients in the Seattle Colon Cancer Family Registry reported that individuals with stage II or III CRC in the highest category of IL-6 concentrations had a significantly higher risk of CRC-specific mortality (HR = 5.02, 95% CI: 2.92–8.59) compared to those with the lowest levels, with this association persisting over 10 years of follow-up [67]. For the soluble IL-6 receptor, the literature is more limited, with one study having found that high expression of sIL6-RA in the tumor epithelium was associated with reduced cancer-specific survival in patients with right-sided colon cancer [68]. However, these observational findings are based on cytokine concentrations measured after cancer diagnosis and treatment, which are likely influenced by factors such as tumor burden, systemic inflammation, and recent chemotherapy or radiotherapy. These post-treatment levels may not reflect pre-diagnostic or causal cytokine activity. In addition, most observational studies assess overall survival, which includes deaths from causes unrelated to CRC, limiting comparability with our outcome of CRC-specific mortality. Confounding by clinical characteristics—such as comorbidities, performance status, or extent of disease at diagnosis—may also bias observed associations, as individuals with more advanced or aggressive cancer are more likely to have elevated inflammatory markers and worse outcomes. Although reverse causation is less likely when the outcome is mortality, elevated cytokine levels may still reflect an inflammatory response to advanced disease or treatment-related complications, or be a contributing cause of reduced survival. In contrast, our MR analysis used genetic variants as proxies for long-term, pre-diagnostic cytokine signaling and evaluated whether a lifelong predisposition to higher IL-6 signaling influences CRC-specific mortality. We observed only modest associations of IL-6 signaling with CRC-specific mortality. A recent MR study from the GECCO consortium found no association between genetically predicted C-reactive protein (CRP) levels and CRC-specific survival [31]. These findings underscore an important biological distinction between upstream cytokine signaling and downstream inflammatory markers. CRP is a hepatic acute-phase protein induced by IL-6 and reflects a general systemic inflammatory response [69], whereas our analyses targeted components of the IL-6 signaling pathway itself. Taken together, these results suggest that while CRP as a downstream unspecific marker of inflammation may not influence CRC-specific prognosis, proximal IL-6–mediated signaling processes could still play a limited role.

To further interpret these findings, we performed colocalization analyses for sIL6-RA and IL6ST, but found little evidence for a shared causal variant with CRC-specific mortality. This suggests that the genetic variants influencing circulating IL-6 pathway proteins may not be the same variants affecting survival in CRC. One possible explanation is that protein QTLs identified in population-based studies do not capture genetic regulation of IL-6 signaling within the tumor microenvironment, where local cytokine activity in epithelial, stromal, or immune cells may be more relevant for disease progression. Another possibility is that these regions contain multiple causal variants or variants acting through closely linked genes, which can make shared signals difficult to detect. In addition, limited power of survival GWAS may reduce the ability to identify colocalization. Therefore, while the colocalization results limit strong locus-specific inference, they do not exclude a role for IL-6 signaling in CRC progression.

Several observational studies have also reported elevated TNF-α levels in individuals with CRC compared to healthy controls [34, 37, 41]. However, findings regarding its association with prognosis are mixed. Two studies reported significantly poorer overall survival in people with CRC who had higher TNF-α levels [34, 41]. A third study by Olsen et al. [40] found that individuals in the highest tertile of TNF-α did not have significantly higher CRC-specific mortality in univariate analysis compared to those in the lowest tertile (HR = 1.5; 95% CI: 0.7–3.0), but the association became statistically significant after adjustment for clinical covariates (HR = 2.3; 95% CI: 1.0–5.4). For TNF receptors, literature on their prognostic value in CRC is scarce. However, in the study by Babic et al. [70], higher pre-diagnostic sTNF-R2 levels were not associated with CRC-specific mortality (HR for highest vs lowest quartile = 1.23; 95% CI, 0.72–2.08). These mixed findings, together with the lack of associations in our MR analyses, underline the complexity of TNF-α as a biomarker and highlight the need for further research to clarify its role in CRC prognosis.

The potential biological mechanisms linking IL-6 and TNF-α to CRC mortality include chronic inflammation, immune modulation [71], and promotion of tumor growth and metastasis through pathways such as cell proliferation [72] and angiogenesis [73]. Although we found little evidence that these genetically predicted cytokine levels influence CRC-specific mortality, the modest association observed for IL-6 signaling via sIL6-RA suggests that this pathway may still play a role. Clinically, IL-6 and TNF-α may still be useful as indicators of disease activity or inflammation, rather than as direct causes of poor outcomes. Such use would relate to cytokine levels measured after diagnosis, and further studies using observed biomarker data are needed to determine their relevance in this context.

### Limitations and strengths

The main limitation of our study is the limited number of SNPs available for TNF-α and its soluble receptors. This resulted in low statistical power, and sometimes wide confidence intervals, and restricted our ability to perform formal collider bias adjustments, which require a large set of well-powered instruments. To address this, we implemented a simulation approach; however, as highlighted by Mitchell et al. [51], simulated data may not fully reflect reality and can be misleading, with no formal recommendations on how to directly compare simulated with observed effects. Another potential limitation is that the genetic associations with cytokine levels used as instruments were derived from general population cohorts, whereas our outcome was CRC-specific mortality among diagnosed patients. It is possible that disease status or treatment could alter cytokine regulation, meaning that the SNP–cytokine effects in patients with CRC may differ from those in the general population, potentially introducing bias. More generally, as with all MR studies, uncertainties remain regarding potential violations of the underlying assumptions, such as unrecognized pleiotropy or weak instrument bias, which cannot be fully excluded despite extensive sensitivity analyses. In addition, pleiotropy-robust methods that require independent instruments (weighted median and MR-PRESSO) could not be applied for most biomarkers because our primary analyses relied on correlated cis-acting variants. However, in cis-MR settings, weighted median approaches are typically underpowered due to the limited number of variants within a locus and the main role of MR-PRESSO—identification of influential outlier variants—was partly addressed through leave-one-out analyses, which did not indicate that any single SNP was driving the observed associations. Together with the use of biologically informed cis-instruments and consistency across two independent GWAS sources, this suggests that major outlier-driven pleiotropy is unlikely. Finally, the generalizability of our findings is currently limited to populations of European ancestry, and to genetic proxies of chronic cytokine exposure rather than short-term, treatment-related fluctuations. Future studies in more diverse populations and with time-specific biomarker measurements are warranted to fully characterize these associations. Despite these limitations, our study has notable strengths. To our knowledge, this is the first MR analysis to investigate associations between genetically predicted IL-6 and TNF-α signaling and CRC-specific mortality. Moreover, the use of MR study design allows us to reduce the possibility of confounding and reverse causation.

## Conclusions

In conclusion, this MR study found that genetically predicted sIL6-RA was weakly associated with increases in CRC-specific mortality, while IL6ST showed associations restricted to stages 2 and 3 disease. We observed no associations for TNF-α, or its soluble receptors. These findings suggest that IL-6 signaling may play a role in CRC progression, although of limited magnitude, whereas TNF-related pathways appear less relevant for prognosis. The complexity of inflammatory signaling in cancer highlights the need for larger, well-powered studies, ideally incorporating diverse populations and time-specific biomarker data, to further clarify the causal role of cytokine pathways and their potential as therapeutic targets in colorectal cancer.

## Supplementary Information

Additional file 1: Tables 1–2. Table 1 Summary information for SNPs used as genetic instruments for sIL6-RA, IL6ST, TNF, sTNF-R1, and sTNF-R2 in Mendelian randomization. Table 2 Genetic summary data for colorectal cancer mortality.

Additional file 2: Figs. 1–17. Figure 1 Two-sample Mendelian randomization (MR) analysis. Figure 2 MR estimates of genetically predicted sIL6R-α on CRC-specific mortality across subgroups in two GWAS. Figure 3 Leave-one-out MR analysis of genetically predicted sIL6R-α and CRC-specific mortality using deCODE GWAS. Figure 4 Leave-one-out MR analysis of genetically predicted sIL6R-α and CRC-specific mortality using UKBB GWAS. Figure 5 MR estimates of genetically predicted IL6ST on CRC-specific mortality across subgroups in two GWAS. Figure 6 Leave-one-out MR analysis of genetically predicted IL6ST and CRC-specific mortality using deCODE GWAS. Figure 7 Leave-one-out MR analysis of genetically predicted IL6ST and CRC-specific mortality using UKBB GWAS. Figure 8 MR estimates of genetically predicted TNF-α on CRC-specific mortality across subgroups using UKBB GWAS. Figure 9 Leave-one-out MR analysis of genetically predicted TNF-α and CRC-specific mortality using UKBB GWAS. Figure 10 MR estimates of genetically predicted sTNF-R1 on CRC-specific mortality across subgroups using deCODE GWAS. Figure 11 Leave-one-out MR analysis of genetically predicted sTNF-R1 and CRC-specific mortality using deCODE GWAS. Figure 12 MR estimates of genetically predicted sTNF-R2 on CRC-specific mortality across subgroups in two GWAS. Figure 13 Leave-one-out MR analysis of genetically predicted sTNF-R2 and CRC-specific mortality using deCODE GWAS. Figure 14 Leave-one-out MR analysis of genetically predicted sTNF-R2 and CRC-specific mortality using UKBB GWAS. Figure 15 Illustration of collider bias in MR analysis. Figure 16 Distribution of simulated MR hazard ratio estimates of TNF-α on CRC-specific mortality. Figure 17 Distribution of the number of SNPs showing spurious association with BMI across 1000 simulations.

Additional file 3: Tables 1–6. Table 1 Minimal detectable hazard ratios (HRs) with 80% power for varying *R*^2^. Table 2 MR estimates of genetically predicted sIL-6RA on CRC-specific mortality using the weighted median method across two GWAS. Table 3 MR-PRESSO results for genetically predicted sIL-6RA and CRC-specific mortality using two GWAS. Table 4 Heterogeneity and pleiotropy tests for MR analyses of genetically predicted biomarkers and CRC-specific mortality. Table 5 Colocalization of sIL-6RA and IL6ST variants with CRC-specific mortality. Table 6 Performance of MR estimation of TNF-α on CRC-specific mortality across 1000 simulations (collider bias simulation).

Additional file 4: STROBE-MR checklist. Checklist of recommended items to address in reports of Mendelian randomization studies.

Additional file 5: GECCO funding statement.

## Data Availability

The summary statistics used in this study for all MR analyses are provided in Additional file 1 for replication purposes. The summary-level GWAS data on outcomes used in this study are available following an application to the Genetics and Epidemiology of Colorectal Cancer Consortium (GECCO).

## Abbreviations

IL-6: Interleukin-6
TNF-α: Tumor necrosis factor-alpha
sIL6-RA: Soluble interleukin-6 receptor alpha
IL6ST: IL-6 signal transducer gp130
sTNF-R1: Soluble tumor necrosis factor receptor one
sTNF-R2: Soluble tumor necrosis factor receptor two
CRP: C-reactive protein
BMI: Body mass index
CRC: Colorectal cancer
IV: Instrumental variable
SNP: Single nucleotide polymorphism
MR: Mendelian randomization
LD: Linkage disequilibrium
GWAS: Genome-wide association study
EAF: Effect allele frequency
SE: Standard error
IVW: Inverse variance weighted
HR: Hazard ratio
SD: Standard deviation
CI: Confidence interval
LOO: Leave-one-out
GECCO: Genetics and Epidemiology of Colorectal Cancer Consortium
