# Supplementary material for: Associations of genetically predicted interleukin-6 and tumor necrosis factor signaling pathways with mortality among persons with colorectal cancer: a two-sample Mendelian randomization
Source: BMC Med. 2026 Mar 6;24:211. doi: 10.1186/s12916-026-04736-9 (PMC13063702; doi:10.1186/s12916-026-04736-9)
Supplement: Supplementary file 2 — Additional file 2: Figs. 1–17. Figure 1 Two-sample Mendelian randomization (MR) analysis. Figure 2 MR estimates of genetically predicted sIL6R-α on CRC-specific mortality across subgroups in two GWAS. Figure 3 Leave-one-out MR analysis of genetically predicted sIL6R-α and CRC-specific mortality using deCODE GWAS. Figure 4 Leave-one-out MR analysis of genetically predicted sIL6R-α and CRC-specific mortality using UKBB GWAS. Figure 5 MR estimates of genetically predicted IL6ST on CRC-specific mortality across subgroups in two GWAS. Figure 6 Leave-one-out MR analysis of genetically predicted IL6ST and CRC-specific mortality using deCODE GWAS. Figure 7 Leave-one-out MR analysis of genetically predicted IL6ST and CRC-specific mortality using UKBB GWAS. Figure 8 MR estimates of genetically predicted TNF-α on CRC-specific mortality across subgroups using UKBB GWAS. Figure 9 Leave-one-out MR analysis of genetically predicted TNF-α and CRC-specific mortality using UKBB GWAS. Figure 10 MR estimates of genetically predicted sTNF-R1 on CRC-specific mortality across subgroups using deCODE GWAS. Figure 11 Leave-one-out MR analysis of genetically predicted sTNF-R1 and CRC-specific mortality using deCODE GWAS. Figure 12 MR estimates of genetically predicted sTNF-R2 on CRC-specific mortality across subgroups in two GWAS. Figure 13 Leave-one-out MR analysis of genetically predicted sTNF-R2 and CRC-specific mortality using deCODE GWAS. Figure 14 Leave-one-out MR analysis of genetically predicted sTNF-R2 and CRC-specific mortality using UKBB GWAS. Figure 15 Illustration of collider bias in MR analysis. Figure 16 Distribution of simulated MR hazard ratio estimates of TNF-α on CRC-specific mortality. Figure 17 Distribution of the number of SNPs showing spurious association with BMI across 1000 simulations. [file 12916_2026_4736_MOESM2_ESM.pdf]

Additional file 2

Additional figures

Title: Associations of genetically predicted interleukin-6 and tumor necrosis factor signaling pathways with mortality among persons with colorectal cancer: A two-sample Mendelian Randomization

Contents

Additional Figure 1: Two-sample Mendelian randomization (MR) analysis.

Additional Figure 2: MR estimates of genetically predicted sIL6R-α on CRC-specific mortality across subgroups in two GWAS.

Additional Figure 3: Leave-one-out MR analysis of genetically predicted sIL6R-α and CRC-specific mortality using deCODE GWAS.

Additional Figure 4: Leave-one-out MR analysis of genetically predicted sIL6R-α and CRC-specific mortality using UKBB GWAS.

Additional Figure 5: MR estimates of genetically predicted IL6ST on CRC-specific mortality across subgroups in two GWAS.

Additional Figure 6: Leave-one-out MR analysis of genetically predicted IL6ST and CRC-specific mortality using deCODE GWAS.

Additional Figure 7: Leave-one-out MR analysis of genetically predicted IL6ST and CRC-specific mortality using UKBB GWAS.

Additional Figure 8: MR estimates of genetically predicted TNF-α on CRC-specific mortality across subgroups using UKBB GWAS.

Additional Figure 9: Leave-one-out MR analysis of genetically predicted TNF-α and CRC-specific mortality using UKBB GWAS.

Additional Figure 10: MR estimates of genetically predicted sTNF-R1 on CRC-specific mortality across subgroups using deCODE GWAS.

Additional Figure 11: Leave-one-out MR analysis of genetically predicted sTNF-R1 and CRC-specific mortality using deCODE GWAS.

Additional Figure 12: MR estimates of genetically predicted sTNF-R2 on CRC-specific mortality across subgroups in two GWAS.

Additional Figure 13: Leave-one-out MR analysis of genetically predicted sTNF-R2 and CRC-specific mortality using deCODE GWAS.

Additional Figure 14: Leave-one-out MR analysis of genetically predicted sTNF-R2 and CRC-specific mortality using UKBB GWAS.

Additional Figure 15: Illustration of Collider Bias in MR Analysis.

Additional Figure 16: Distribution of Simulated MR Hazard Ratio Estimates of TNF-α on CRC-Specific Mortality.

Additional Figure 17: Distribution of number of SNPs showing spurious association with BMI across 1,000 simulations.

Additional Figure 1: Two-sample Mendelian randomization (MR) analysis.

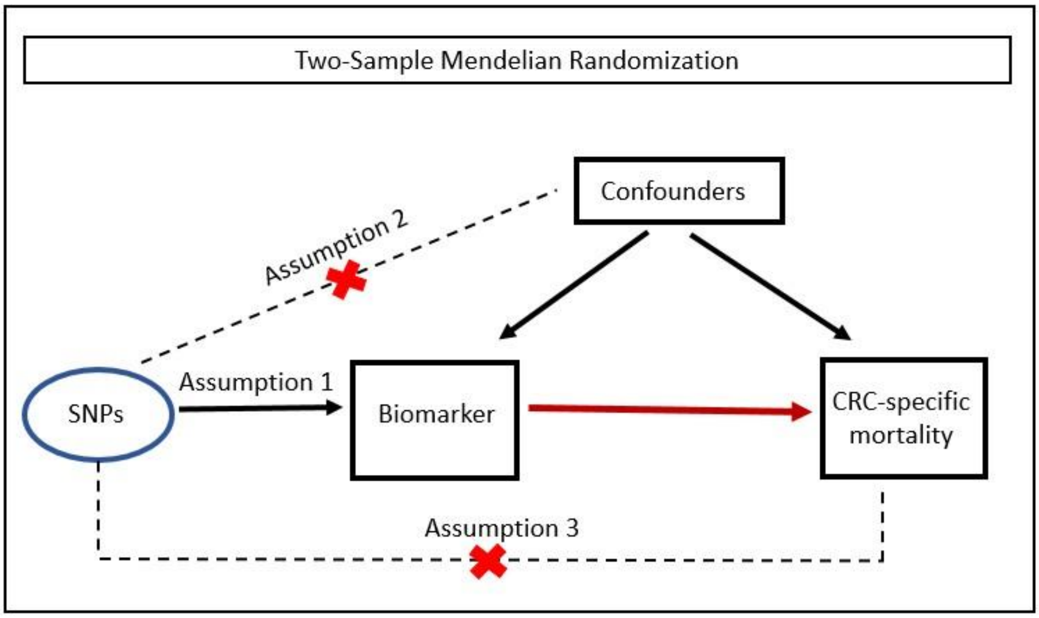

[a]SNPs are used as instrumental variables to estimate the association between IL-6 or TNF-α and colorectal cancer-specific (CRC) mortality. The three core MR assumptions are illustrated: (1) the genetic variants (SNPs) are associated with the exposure (IL-6 or TNF-α); (2) the SNPs are not associated with confounders of the exposure-outcome relationship; and (3) the SNPs influence the outcome (CRC mortality) only through the exposure and not via alternative pathways. Red crosses indicate potential violations of assumptions 2 and 3.

Additional Figure 2: MR estimates of genetically predicted sIL6R-α on CRC-specific mortality across subgroups in two GWAS.

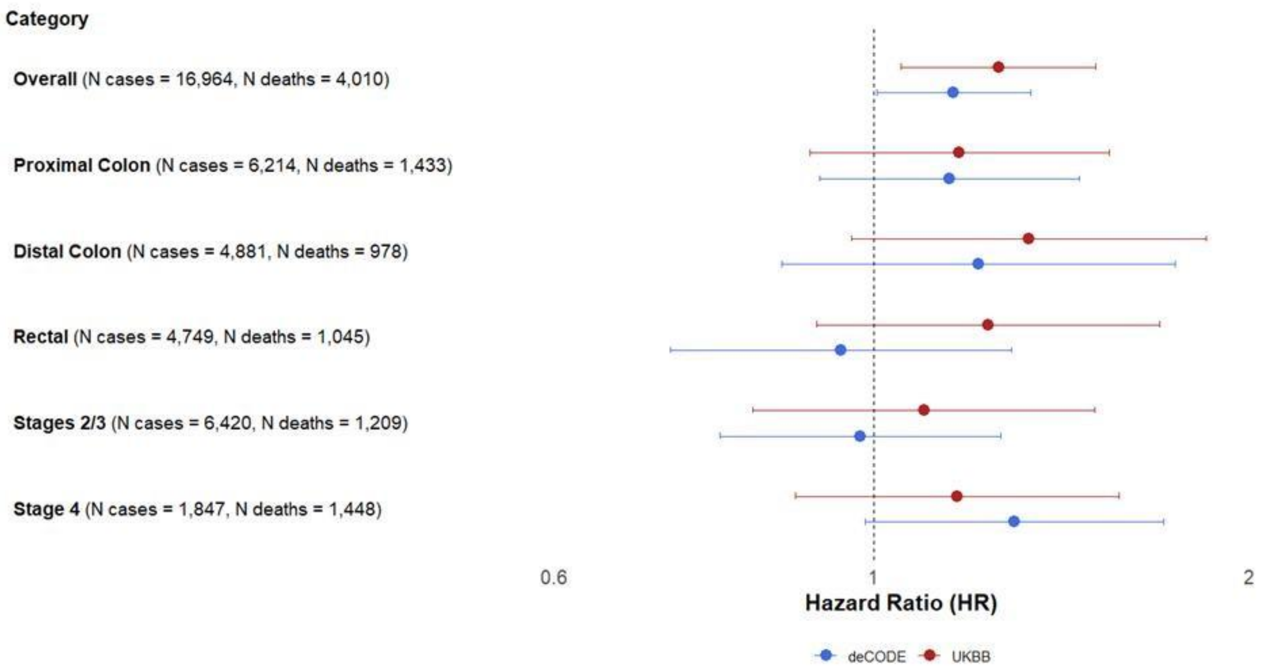

[a]Hazard ratios (HRs) and 95% confidence intervals (CIs) are presented for the estimated effects of genetically predicted inflammatory biomarker on colorectal cancer-specific mortality, using Mendelian Randomization (MR) with the Inverse Variance Weighted (IVW) method.

[b]Genetic cis instruments for biomarker levels were derived from two genome-wide association studies (GWAS): deCODE genetics (Ferkingstad et al., 2021, (1)) and UK Biobank (UKBB) (Sun et al., 2023 (2)). [c]HRs represent the effect per one standard deviation (SD) increase in biomarker levels.

Additional Figure 3: Leave-one-out MR analysis of genetically predicted sIL6R-α and CRC-specific mortality using deCODE GWAS.

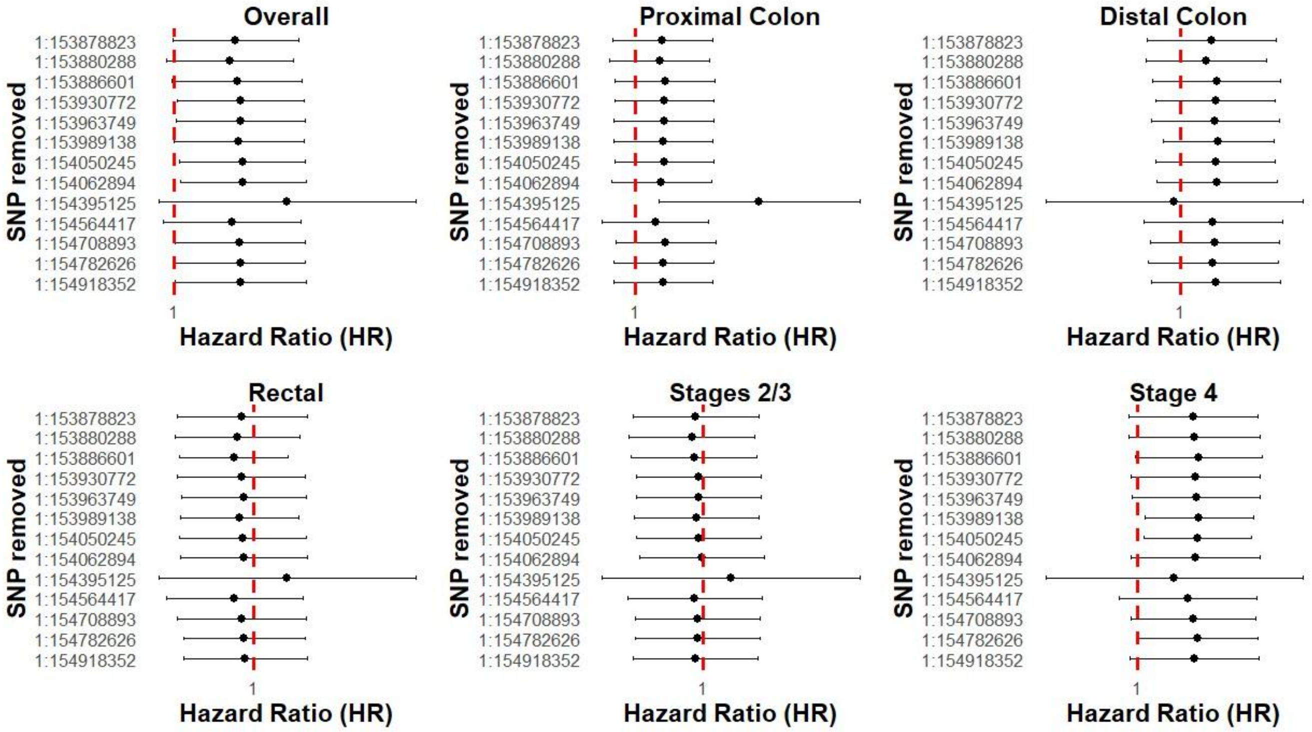

[a]Hazard ratios (HRs) and 95% confidence intervals (CIs) are presented for the estimated effects of genetically predicted inflammatory biomarker on colorectal cancer-specific mortality, using Mendelian Randomization (MR) with the Inverse Variance Weighted (IVW) method.

[b]Genetic cis instruments for biomarker levels were derived from the genome-wide association study (GWAS) deCODE genetics (Ferkingstad et al., 2021).

[c]HRs represent the effect per one standard deviation (SD) increase in biomarker levels.

Additional Figure 4: Leave-one-out MR analysis of genetically predicted sIL6R-α and CRC-specific mortality using UKBB GWAS.

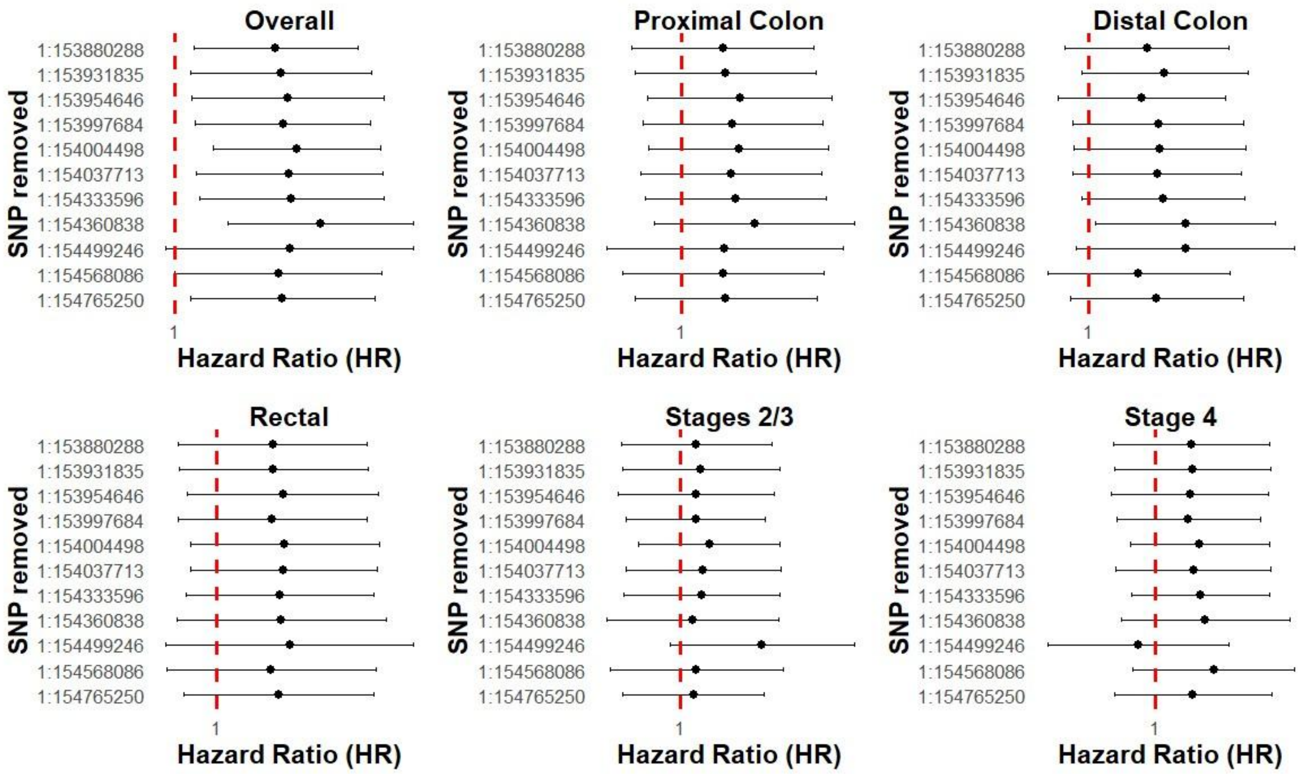

[a]Hazard ratios (HRs) and 95% confidence intervals (CIs) are presented for the estimated effects of genetically predicted inflammatory biomarker on colorectal cancer-specific mortality, using Mendelian Randomization (MR) with the Inverse Variance Weighted (IVW) method.

[b]Genetic cis instruments for biomarker levels were derived from the genome-wide association study (GWAS) UK Biobank (UKBB), (Sun et al., 2023).

[c]HRs represent the effect per one standard deviation (SD) increase in biomarker levels.

Additional Figure 5: MR estimates of genetically predicted IL6ST on CRC-specific mortality across subgroups in two GWAS.

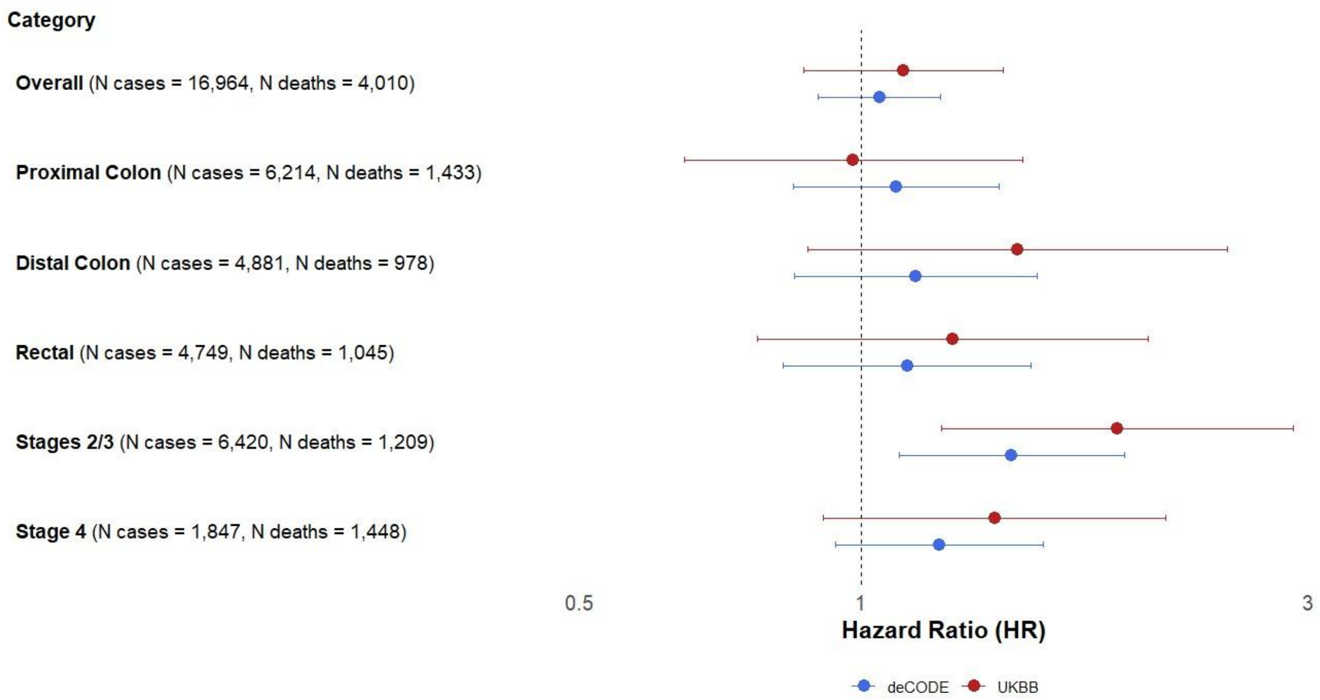

[a]Hazard ratios (HRs) and 95% confidence intervals (CIs) are presented for the estimated effects of genetically predicted inflammatory biomarker on colorectal cancer-specific mortality, using Mendelian Randomization (MR) with the Inverse Variance Weighted (IVW) method.

[b]Genetic cis instruments for biomarker levels were derived from two genome-wide association studies (GWAS): deCODE genetics (Ferkingstad et al., 2021) and UK Biobank (UKBB) (Sun et al., 2023). [c]HRs represent the effect per one standard deviation (SD) increase in biomarker levels.

Additional Figure 6: Leave-one-out MR analysis of genetically predicted IL6ST and CRC-specific mortality using deCODE GWAS.

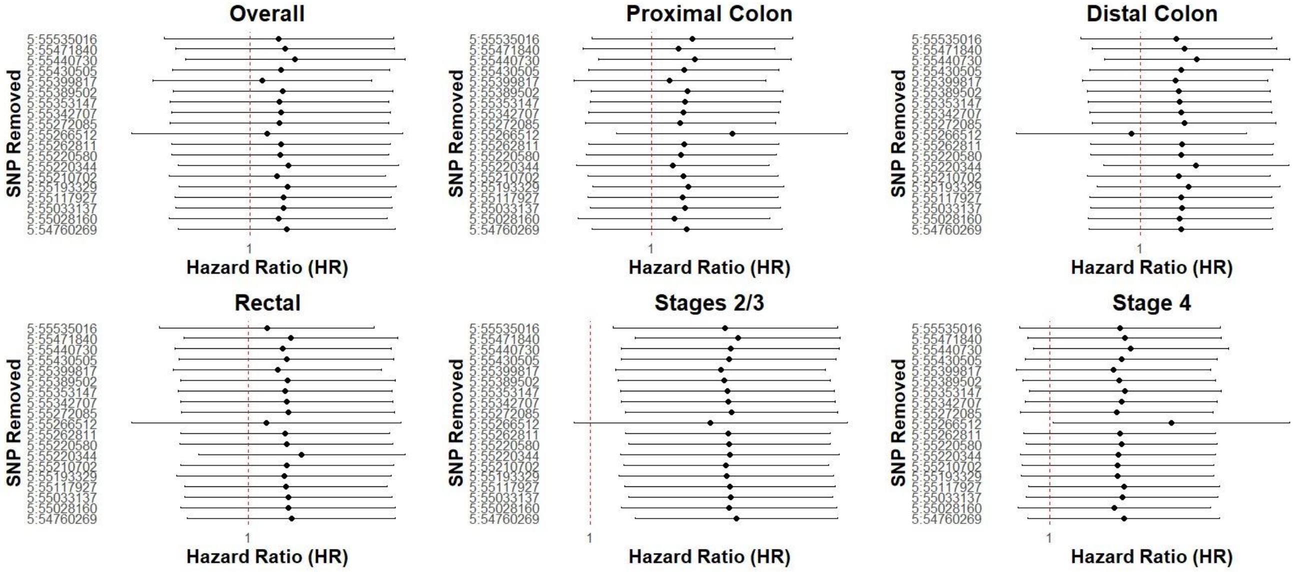

[a]Hazard ratios (HRs) and 95% confidence intervals (CIs) are presented for the estimated effects of genetically predicted inflammatory biomarker on colorectal cancer-specific mortality, using Mendelian Randomization (MR) with the Inverse Variance Weighted (IVW) method.

[b]Genetic cis instruments for biomarker levels were derived from the genome-wide association study (GWAS) deCODE genetics (Ferkingstad et al., 2021). [c]HRs represent the effect per one standard deviation (SD) increase in biomarker levels.

Additional Figure 7: Leave-one-out MR analysis of genetically predicted IL6ST and CRC-specific mortality using UKBB GWAS.

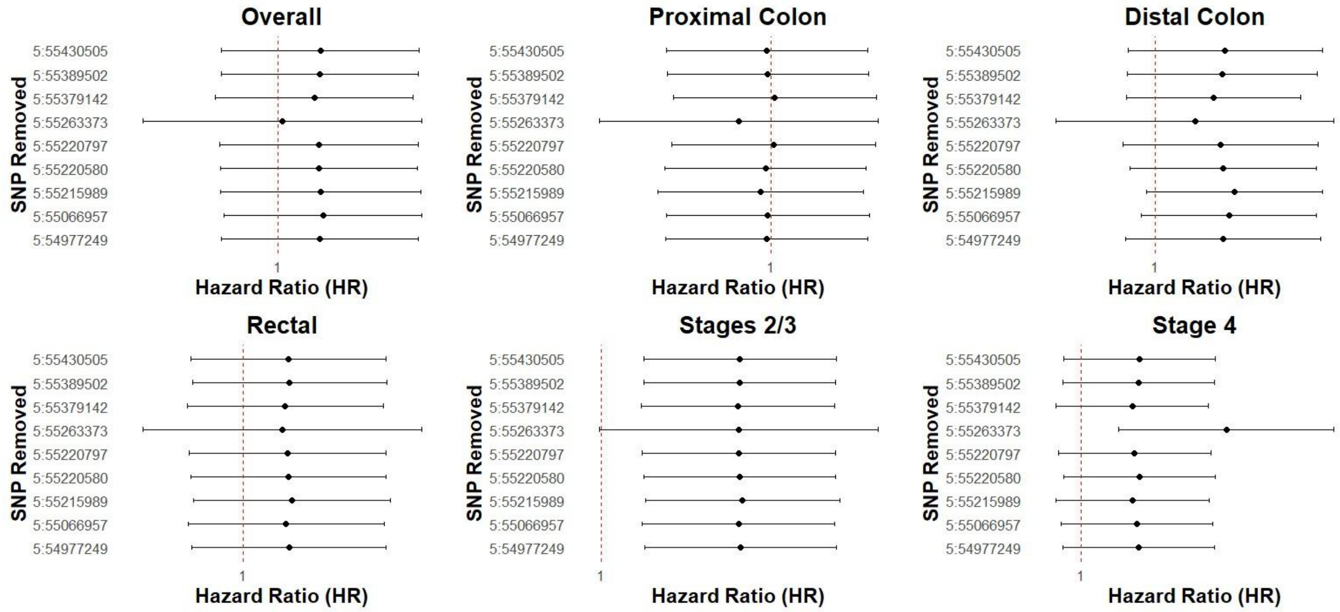

[a]Hazard ratios (HRs) and 95% confidence intervals (CIs) are presented for the estimated effects of genetically predicted inflammatory biomarker on colorectal cancer-specific mortality, using Mendelian Randomization (MR) with the Inverse Variance Weighted (IVW) method.

[b]Genetic cis instruments for biomarker levels were derived from the genome-wide association study (GWAS) UK Biobank (UKBB), (Sun et al., 2023). [c]HRs represent the effect per one standard deviation (SD) increase in biomarker levels.

Additional Figure 8: MR estimates of genetically predicted TNF-α on CRC-specific mortality across subgroups using UKBB GWAS.

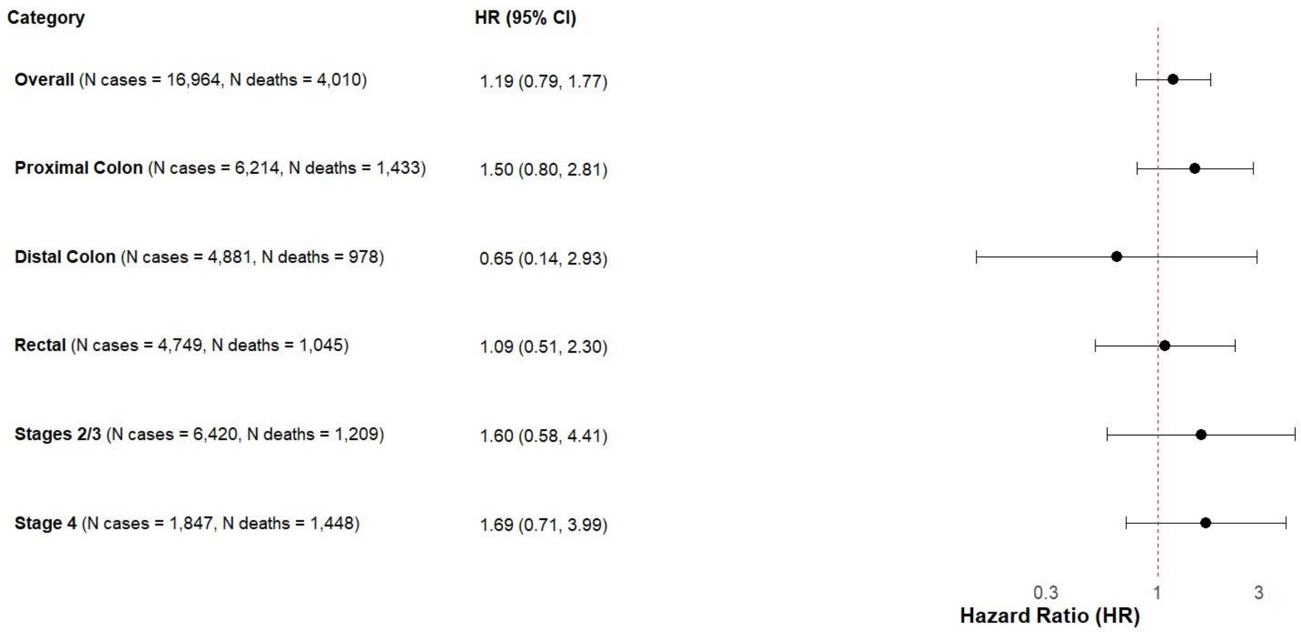

[a]Hazard ratios (HRs) and 95% confidence intervals (CIs) are presented for the estimated effects of genetically predicted inflammatory biomarker on colorectal cancer-specific mortality, using Mendelian Randomization (MR) with the Inverse Variance Weighted (IVW) method.

[b]Genetic cis instruments for biomarker levels were derived from the genome-wide association study (GWAS) UK Biobank (UKBB) (Sun et al., 2023).

[c]HRs represent the effect per one standard deviation (SD) increase in biomarker levels.

Additional Figure 9: Leave-one-out MR analysis of genetically predicted TNF-α and CRC-specific mortality using UKBB GWAS.

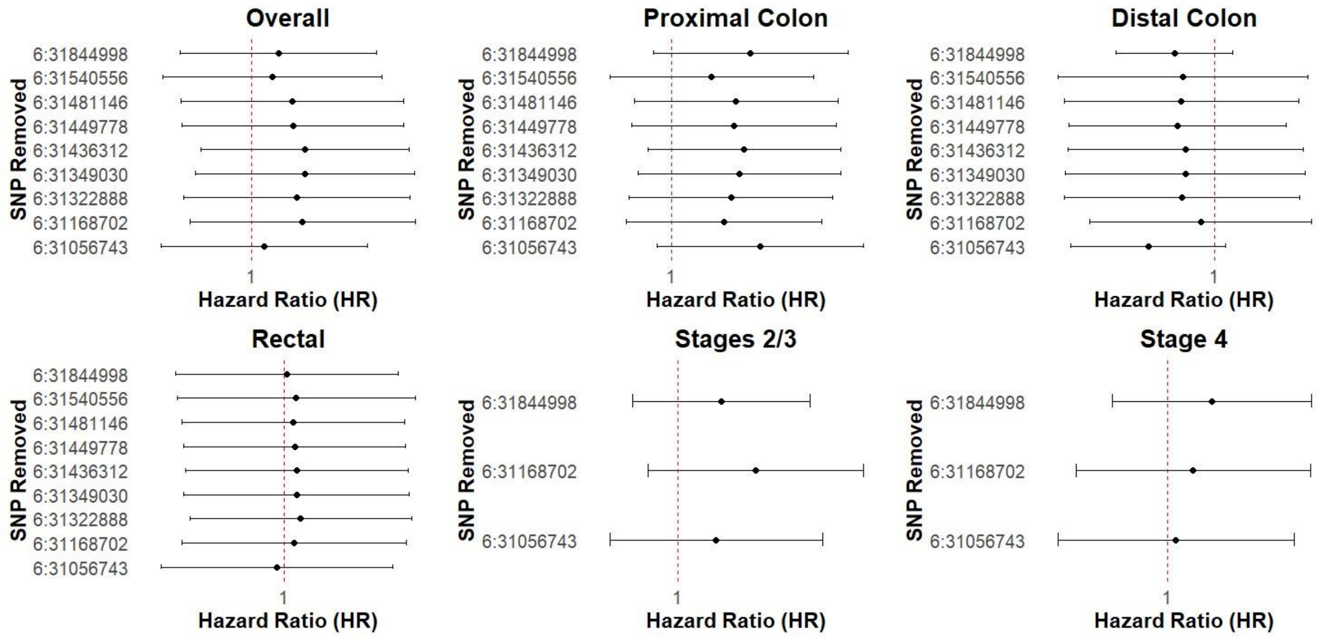

[a]Hazard ratios (HRs) and 95% confidence intervals (CIs) are presented for the estimated effects of genetically predicted inflammatory biomarker on colorectal cancer-specific mortality, using Mendelian Randomization (MR) with the Inverse Variance Weighted (IVW) method.

[b]Genetic cis instruments for biomarker levels were derived from the genome-wide association study (GWAS) UK Biobank (UKBB), (Sun et al., 2023). [c]HRs represent the effect per one standard deviation (SD) increase in biomarker levels.

Additional Figure 10: MR estimates of genetically predicted sTNF-R1 on CRC-specific mortality across subgroups using deCODE GWAS.

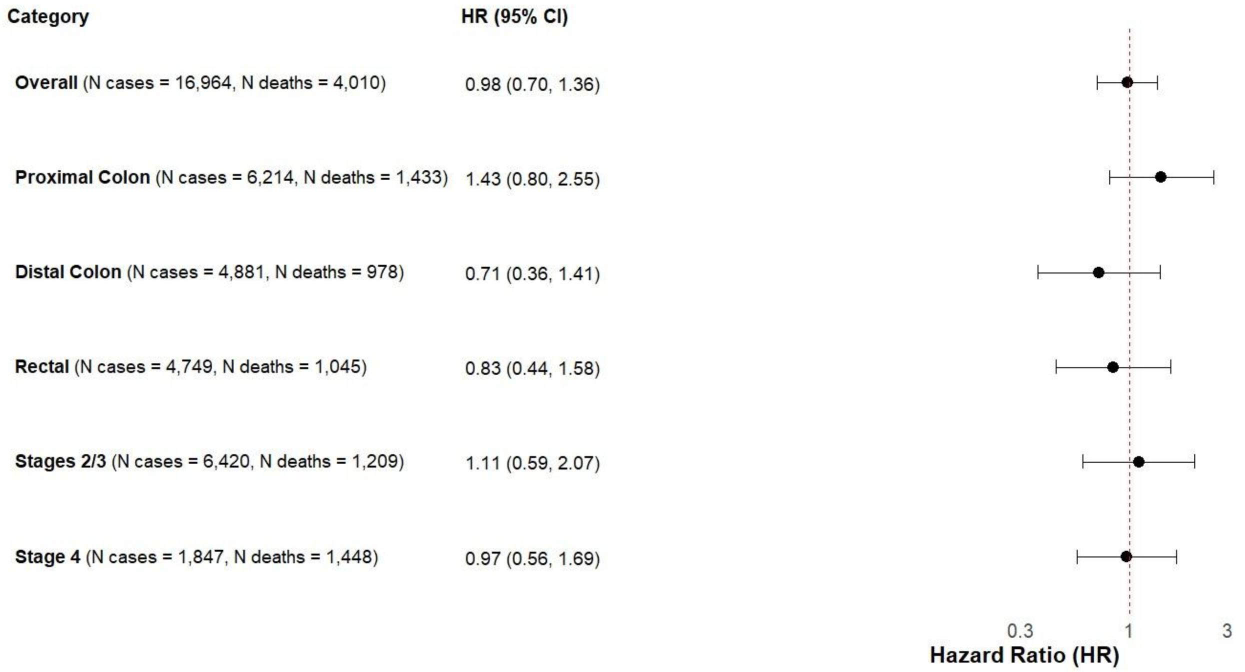

[a]Hazard ratios (HRs) and 95% confidence intervals (CIs) are presented for the estimated effects of genetically predicted inflammatory biomarker on colorectal cancer-specific mortality, using Mendelian Randomization (MR) with the Inverse Variance Weighted (IVW) method.

[b]Genetic cis instruments for biomarker levels were derived from the genome-wide association study (GWAS) deCODE genetics (Ferkingstad et al., 2021).

[c]HRs represent the effect per one standard deviation (SD) increase in biomarker levels.

Additional Figure 11: Leave-one-out MR analysis of genetically predicted sTNF-R1 and CRC-specific mortality using deCODE GWAS.

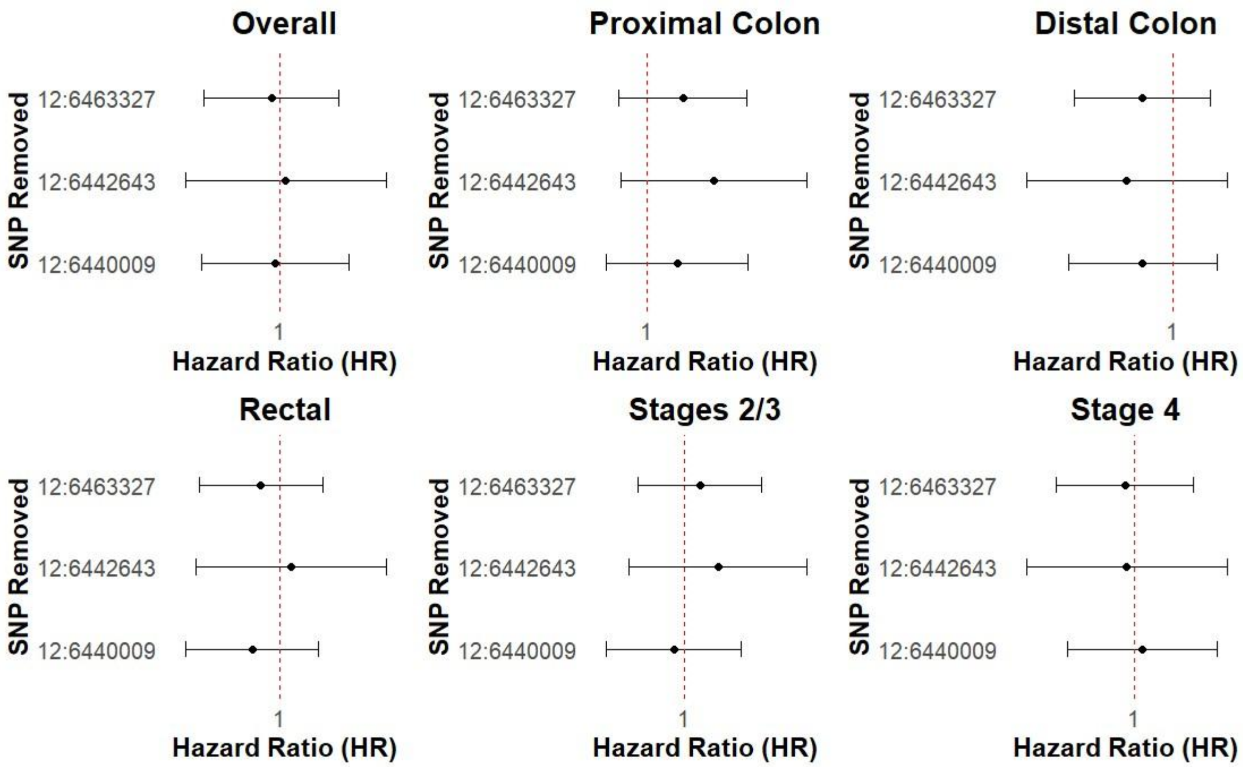

[a]Hazard ratios (HRs) and 95% confidence intervals (CIs) are presented for the estimated effects of genetically predicted inflammatory biomarker on colorectal cancer-specific mortality, using Mendelian Randomization (MR) with the Inverse Variance Weighted (IVW) method.

[b]Genetic cis instruments for biomarker levels were derived from the genome-wide association study (GWAS) deCODE genetics (Ferkingstad et al., 2021).

[c]HRs represent the effect per one standard deviation (SD) increase in biomarker levels.

Additional Figure 12: MR estimates of genetically predicted sTNF-R2 on CRC-specific mortality across subgroups in two GWAS.

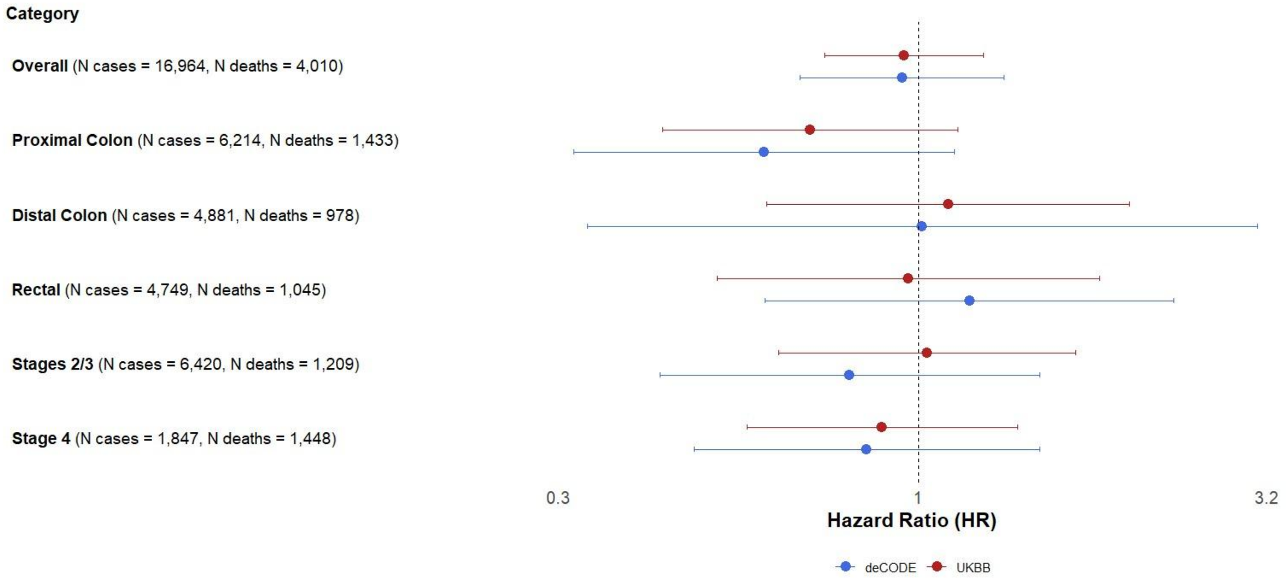

[a]Hazard ratios (HRs) and 95% confidence intervals (CIs) are presented for the estimated effects of genetically predicted inflammatory biomarker on colorectal cancer-specific mortality, using Mendelian Randomization (MR) with the Inverse Variance Weighted (IVW) method.

[b]Genetic cis instruments for biomarker levels were derived from two genome-wide association studies (GWAS): deCODE genetics (Ferkingstad et al., 2021) and UK Biobank (UKBB) (Sun et al., 2023). [c]HRs represent the effect per one standard deviation (SD) increase in biomarker levels.

Additional Figure 13: Leave-one-out MR analysis of genetically predicted sTNF-R2 and CRC-specific mortality using deCODE GWAS.

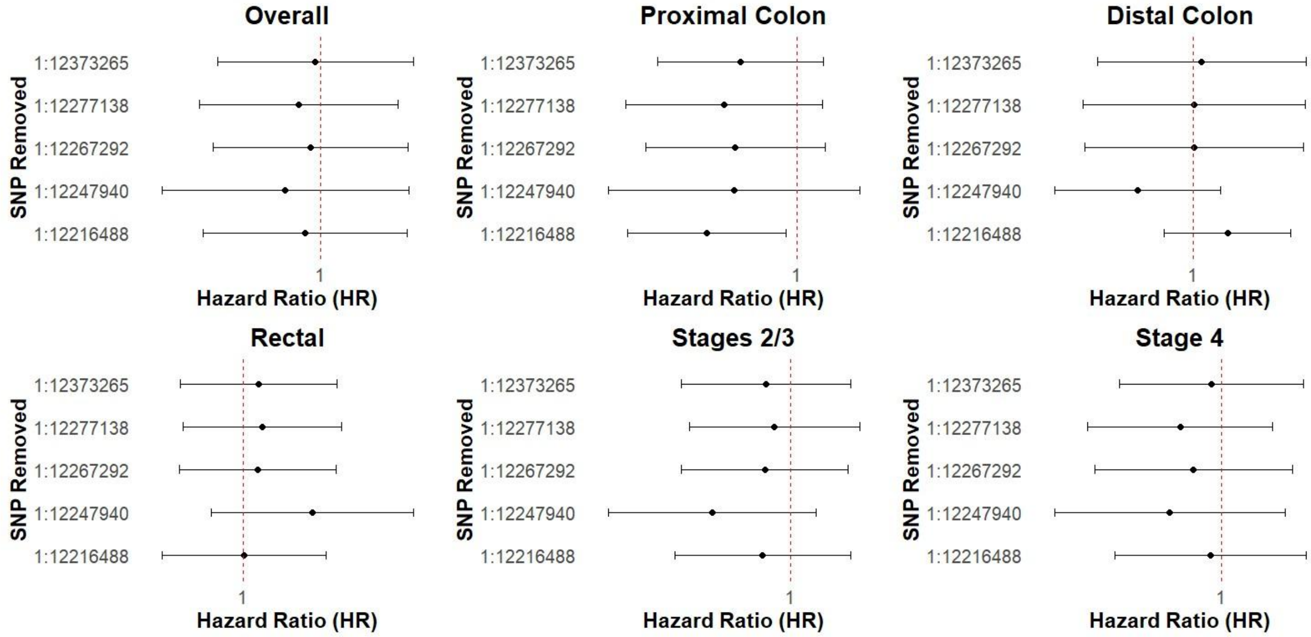

[a]Hazard ratios (HRs) and 95% confidence intervals (CIs) are presented for the estimated effects of genetically predicted inflammatory biomarker on colorectal cancer-specific mortality, using Mendelian Randomization (MR) with the Inverse Variance Weighted (IVW) method.

[b]Genetic cis instruments for biomarker levels were derived from the genome-wide association study (GWAS) deCODE genetics (Ferkingstad et al., 2021). [c]HRs represent the effect per one standard deviation (SD) increase in biomarker levels.

Additional Figure 14: Leave-one-out MR analysis of genetically predicted sTNF-R2 and CRC-specific mortality using UKBB GWAS.

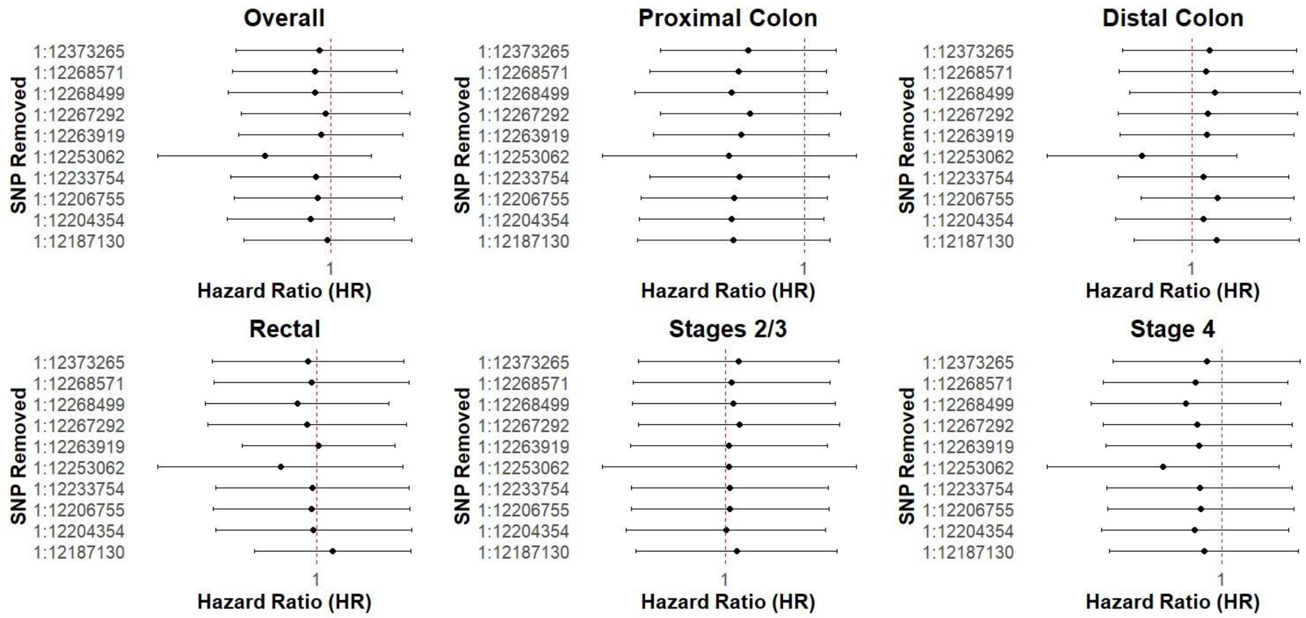

[a]Hazard ratios (HRs) and 95% confidence intervals (CIs) are presented for the estimated effects of genetically predicted inflammatory biomarker on colorectal cancer-specific mortality, using Mendelian Randomization (MR) with the Inverse Variance Weighted (IVW) method.

[b]Genetic cis instruments for biomarker levels were derived from the genome-wide association study (GWAS) UK Biobank (UKBB), (Sun et al., 2023). [c]HRs represent the effect per one standard deviation (SD) increase in biomarker levels.

Additional Figure 15: Illustration of Collider Bias in MR Analysis.

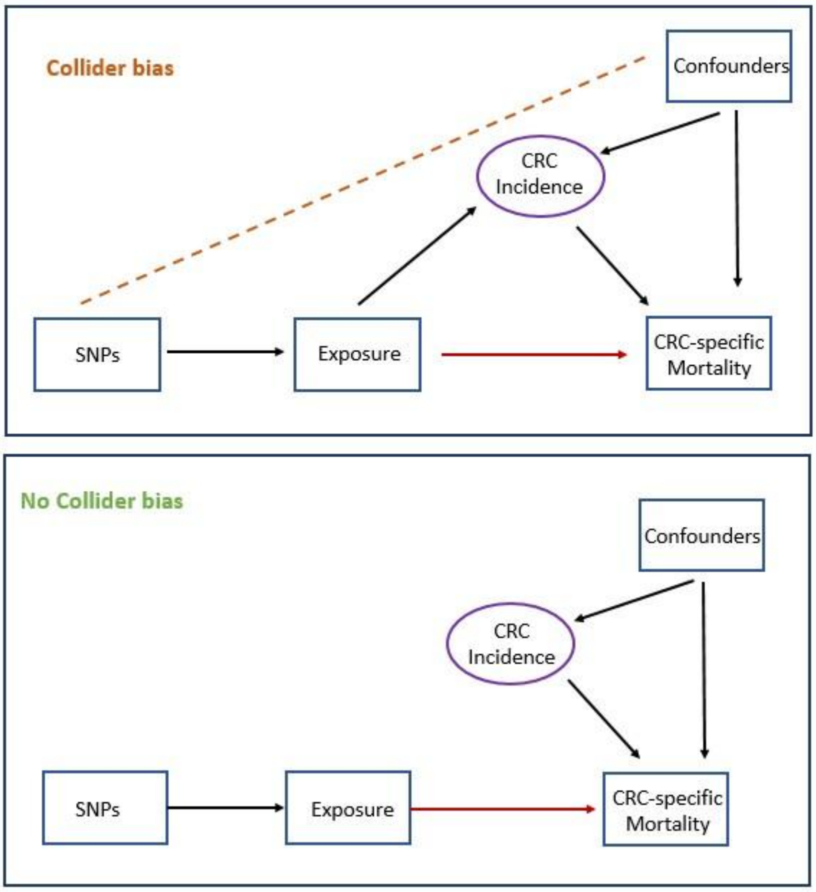

Additional Figure 16: Distribution of Simulated MR Hazard Ratio Estimates of TNF-α on CRC-Specific Mortality.

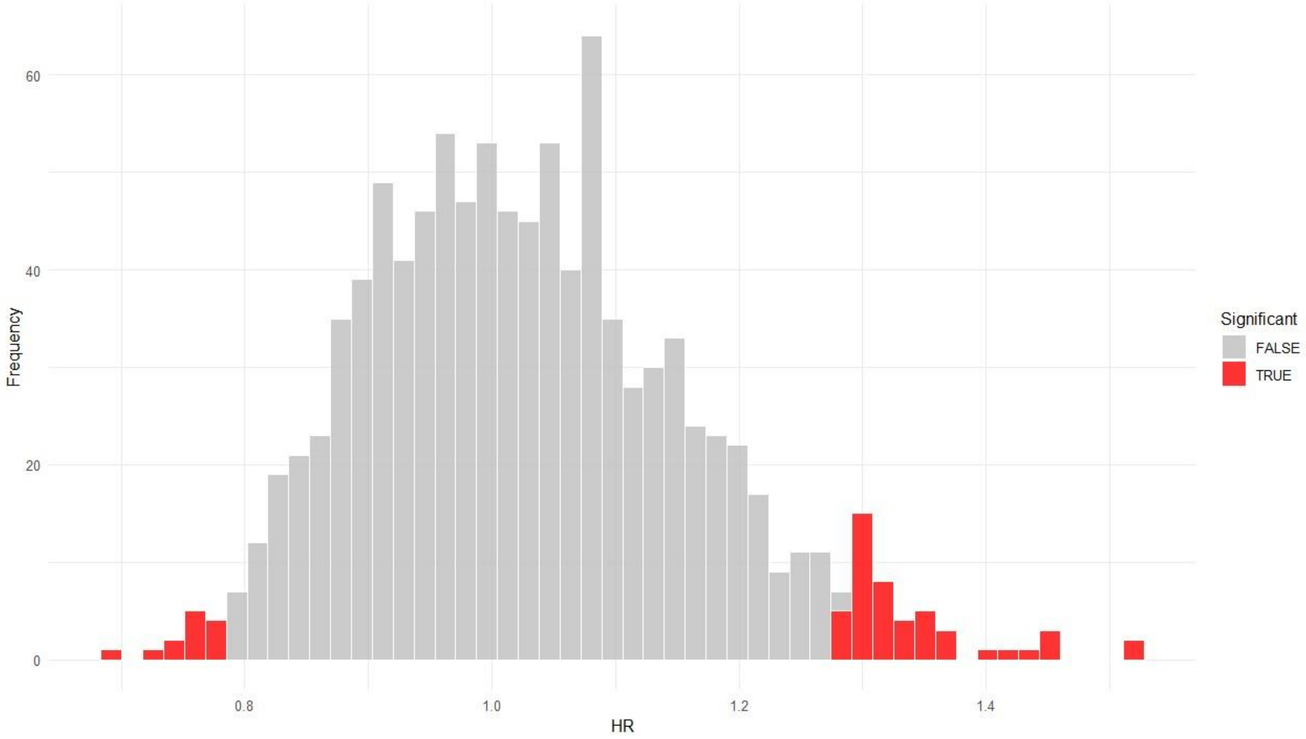

[a]Results are based on 1,000 simulated Mendelian randomization analyses under the null hypothesis of no causal effect of genetically predicted TNF-α on CRC-specific mortality. [b]Bars represent the frequency distribution of Hazard Ratio (HR) estimates across simulations.

[c]Red bars indicate simulations where the MR estimate reached nominal statistical significance ($p < 0.05$); grey bars indicate non-significant estimates.

[d]The distribution illustrates the small upward bias in HR estimates and the modest inflation in type I error due to collider bias when conditioning on CRC incidence.

Additional Figure 17: Distribution of number of SNPs showing spurious association with BMI across 1,000 simulations.

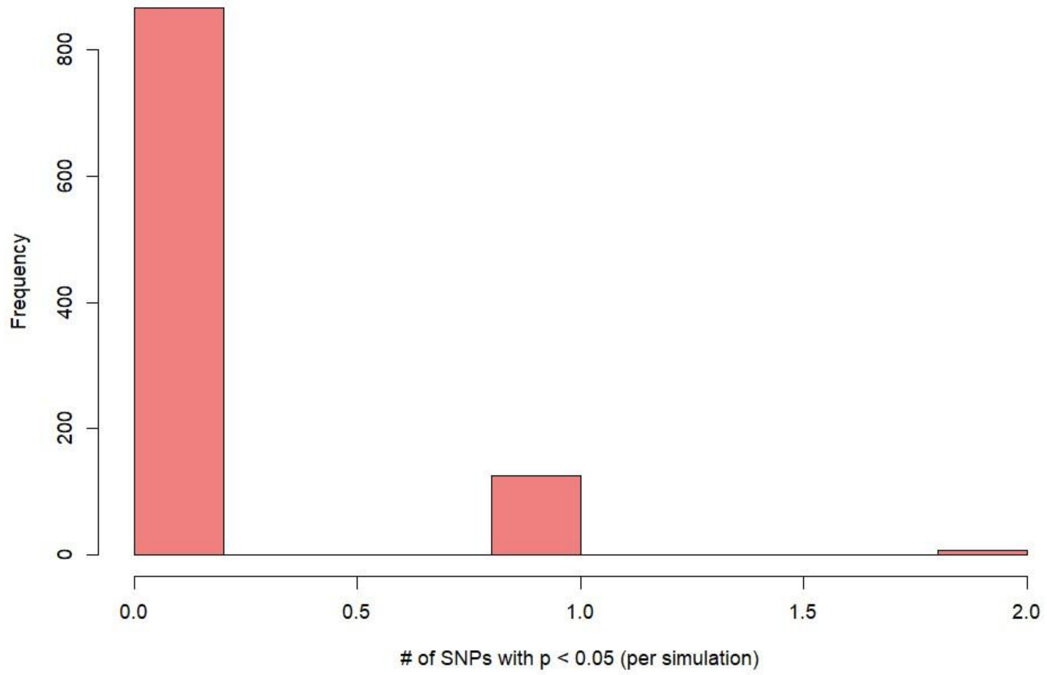

[a]Each bar represents the frequency of simulations in which 0, 1, or 2 SNPs showed a nominal association with BMI after conditioning on CRC incidence.

[b]In ~87% of simulations, no SNPs were spuriously associated with BMI; in ~13% at least one SNP reached nominal significance.

[c]These findings illustrate that collider bias can occasionally induce spurious SNP–confounder associations, though the overall frequency was low.
