# Supplementary material for: Associations of genetically predicted interleukin-6 and tumor necrosis factor signaling pathways with mortality among persons with colorectal cancer: a two-sample Mendelian randomization
Source: BMC Med. 2026 Mar 6;24:211. doi: 10.1186/s12916-026-04736-9 (PMC13063702; doi:10.1186/s12916-026-04736-9)
Supplement: Supplementary file 3 — Additional file 3: Tables 1–6. Table 1 Minimal detectable hazard ratios (HRs) with 80% power for varying R2. Table 2 MR estimates of genetically predicted sIL-6RA on CRC-specific mortality using the weighted median method across two GWAS. Table 3 MR-PRESSO results for genetically predicted sIL-6RA and CRC-specific mortality using two GWAS. Table 4 Heterogeneity and pleiotropy tests for MR analyses of genetically predicted biomarkers and CRC-specific mortality. Table 5 Colocalization of sIL-6RA and IL6ST variants with CRC-specific mortality. Table 6 Performance of MR estimation of TNF-α on CRC-specific mortality across 1000 simulations (collider bias simulation). [file 12916_2026_4736_MOESM3_ESM.pdf]

Additional file 3

Supplementary Tables and Simulation process

Title: Associations of genetically predicted interleukin-6 and tumor necrosis factor signaling pathways with mortality among persons with colorectal cancer: A two-sample Mendelian Randomization

Contents:

Additional Table 1: Minimal Detectable Hazard Ratios (HRs) with a power of 80% for varying R^2.

Additional Table 2: Mendelian Randomization (MR) estimates of genetically predicted sIL-6RA on CRC-specific mortality, with weighted median method using two genome-wide association studies.

Additional Table 3: MR-PRESSO results for genetically predicted sIL-6RA and CRC-specific mortality using two genome-wide association studies.

Additional Table 4: Heterogeneity and Pleiotropy Tests for Mendelian Randomization (MR) of genetically predicted biomarkers and CRC-specific mortality.

Additional Table 5: Colocalization of sIL-6RA and IL6ST variants with CRC-specific mortality.

Collider bias simulation details

Additional Table 6: Performance of Mendelian Randomization (MR) estimation of TNF-α on CRC-specific mortality across 1,000 Simulations

Additional Table 1: Minimal Detectable Hazard Ratios (HRs) with a power of 80% for varying R^2.

	deCODE			UKBB		
	**HR**	**R2**	**nSNP**	**HR**	**R^2**	**nSNP**
sIL6-RA	1.05	49%	13	1.07	25%	11
IL6ST	1.14	6.9%	23	1.21	3.3%	12
TNF-α	NA	NA	NA	1.24	2.6%	14
sTNF-R1	1.42	1%	4	NA	NA	NA
sTNF-R2	1.42	1%	5	1.23	2.8%	11

[a]HRs correspond to a per one standard deviation (SD) increase in genetically predicted biomarker levels.

[b]Genetic cis instruments for biomarker levels were derived from two genome-wide association studies (GWAS): deCODE genetics (Ferkingstad et al., 2021) and UK Biobank (UKBB) (Sun et al., 2023).

[c]nSNP=number of SNPs, NA=Not Applicable

Additional Table 2: Mendelian Randomization (MR) estimates of genetically predicted sIL-6RA on CRC-specific mortality, with weighted median method using two genome-wide association studies.

	deCODE		UKBB		
	**HR (95% CI)**	**nSNP**	**HR (95% CI)**	**nSNP**	**Method**
Overall	1.05 (0.99, 1.12)	13	1.09 (1.01, 1.18)	11	Weighted median
Proximal Colon	1.01 (0.91, 1.11)	13	1.08 (0.95, 1.22)	11	Weighted median
Distal Colon	1.10 (0.98, 1.24)	13	1.09 (0.93, 1.27)	11	Weighted median
Rectal	0.97 (0.87, 1.09)	13	1.07 (0.93, 1.23)	11	Weighted median
Stages 2/3	0.98 (0.89, 1.09)	13	1.05 (0.91, 1.21)	11	Weighted median
Stage 4	1.12 (1.01, 1.24)	13	1.15 (0.99, 1.33)	11	Weighted median

[a]Hazard ratios (HRs) and 95% confidence intervals (CIs) are presented for the estimated effects of genetically predicted inflammatory biomarker on colorectal cancer-specific mortality, using MR with the weighted median method.

[b]Genetic cis instruments for biomarker levels were derived from two GWAS: deCODE genetics (Ferkingstad et al., 2021) and UK Biobank (UKBB) (Sun et al., 2023).

[c]HRs represent the effect per one standard deviation (SD) increase in biomarker levels

Additional Table 3: MR-PRESSO results for genetically predicted sIL-6RA and CRC-specific mortality using two genome-wide association studies.

	**deCODE**	**UKBB**
	**MR PRESSO** (*P*)	**MR PRESSO** (*P*)
Overall	0.56	0.37
Proximal Colon	0.54	0.84
Distal Colon	0.27	0.47
Rectal	0.38	0.90
Stages 2/3	0.83	0.33
Stage 4	0.42	0.33

[a]Genetic cis instruments for biomarker levels were derived from two GWAS: deCODE genetics (Ferkingstad et al., 2021) and UK Biobank (UKBB) (Sun et al., 2023).

Additional Table 4: Heterogeneity and Pleiotropy Tests for Mendelian Randomization (MR) of genetically predicted biomarkers and CRC-specific mortality.

Biomarker	deCODE			UKBB		
	IVW Heterogeneity (*P*)	MR-Egger Heterogeneity (*P*)	Pleiotropy (*P*)	IVW Heterogeneity (*P*)	MR-Egger Heterogeneity (*P*)	Pleiotropy (*P*)
**sIL-6RA**						
Overall	0.45	0.40	0.54	0.29	0.23	0.64
Proximal Colon	0.81	0.87	0.24	0.82	0.76	0.65
Distal Colon	0.08	0.15	0.15	0.44	0.38	0.58
Rectal	0.21	0.17	0.66	0.84	0.77	0.82
Stages 2/3	0.80	0.74	0.93	0.33	0.56	0.09
Stage 4	0.20	0.18	0.51	0.33	0.30	0.49
**IL6ST**						
Overall	0.49	0.47	0.43	0.95	0.91	0.79
Proximal Colon	0.69	0.63	0.88	0.73	0.73	0.38
Distal Colon	0.59	0.81	0.04	0.34	0.25	0.99
Rectal	0.40	0.34	0.84	0.95	0.91	0.90
Stages 2/3	0.38	0.32	0.77	0.92	0.87	0.93
Stage 4	0.62	0.63	0.31	0.57	0.92	0.04
**TNF**						
Overall	NA	NA	NA	0.32	0.25	0.69
Proximal Colon	NA	NA	NA	0.44	0.36	0.59
Distal Colon	NA	NA	NA	0.00	0.00	0.83
Rectal	NA	NA	NA	0.98	0.98	0.56
Stages 2/3	NA	NA	NA	0.42	0.19	0.92
Stage 4	NA	NA	NA	0.05	0.05	0.46
**sTNF-R1**						
Overall	0.85	0.59	0.87	NA	NA	NA
Proximal Colon	0.71	0.69	0.47	NA	NA	NA
Distal Colon	0.92	0.76	0.78	NA	NA	NA
Rectal	0.74	0.97	0.43	NA	NA	NA
Stages 2/3	0.27	0.22	0.40	NA	NA	NA
Stage 4	0.52	0.28	0.73	NA	NA	NA
**sTNF-R2**						
Overall	0.84	0.74	0.67	0.49	0.68	0.09
Proximal Colon	0.27	0.20	0.58	0.24	0.18	0.72
Distal Colon	0.04	0.26	0.04	0.30	0.51	0.06
Rectal	0.56	0.93	0.11	0.16	0.16	0.37
Stages 2/3	0.56	0.46	0.52	0.95	0.92	0.80
Stage 4	0.47	0.37	0.52	0.90	0.89	0.42

[a]IVW Heterogeneity (p-value): Cochran's Q statistic assessing heterogeneity in inverse-variance weighted (IVW) MR estimates.

[b]MR-Egger Heterogeneity (p-value): Heterogeneity test for MR-Egger regression.

[c]Pleiotropy (p-value): MR-Egger intercept test for directional pleiotropy.

[d]NA= Not Applicable.

[e]Genetic cis instruments for biomarker levels were derived from two genome-wide association studies (GWAS): deCODE genetics (Ferkingstad et al., 2021) and UK Biobank (UKBB) (Sun et al., 2023).

Additional Table 5: Colocalization of sIL-6RA and IL6ST variants with CRC-specific mortality.

	deCODE GWAS		UKBB GWAS		
**Posterior**	**Value**	**nSNP**	**Value**	**nSNP**	**Interpretation**
**sIL-6RA**		853		891	
PP.H0	0.00%		0.00%		No association with either trait
PP.H1	92.53%		92.60%		Association with pQTL (sIL6-RA) only
PP.H2	0.00%		0.00%		Association with GWAS (CRC mortality) only
PP.H3	2.85%		2.95%		Both traits associated, but with different causal SNPs
PP.H4	4.62%		4.49%		Colocalization: both traits share the same causal SNP
**IL6ST**				905	
PP.H0	0.00%		0.00%		No association with either trait
PP.H1	93.40%		93.22%		Association with pQTL (sIL6-RA) only
PP.H2	0.00%		0.00%		Association with GWAS (CRC mortality) only
PP.H3	4.32%		4.52%		Both traits associated, but with different causal SNPs
PP.H4	2.28%		2.25%		Colocalization: both traits share the same causal SNP

[a]Genetic cis instruments for biomarker levels were derived from two genome-wide association studies (GWAS): deCODE genetics (Ferkingstad et al., 2021) and UK Biobank (UKBB) (Sun et al., 2023).

Collider bias simulation

Goal and rationale

The objective of the simulation was to assess whether collider bias could generate spurious associations in our MR analysis of genetically predicted TNF-α and CRC-specific mortality, and if so, to approximate the magnitude of such bias. This approach was adapted from Noyce et al. (2017) and builds on ideas presented by Mitchell et al. (2022). Collider bias arises in MR studies of survival when analyses are restricted to cases, and exposures (or their genetic instruments) are also associated with disease incidence, potentially inducing associations between the instruments and confounders. Because TNF-α has been linked to CRC incidence, this analysis was susceptible to collider bias.

In our simulations, SNPs were set to influence TNF levels. CRC incidence was modeled as a function of genetically predicted TNF, BMI (chosen as a representative confounder, although other confounders could have been selected), and age. CRC mortality was modeled as a function of BMI and age. Age was included to reflect its established influence on both incidence and survival, introducing a realistic time component. After generating the data, we examined whether spurious associations between TNF and CRC-specific mortality emerged under the null, and whether TNF SNPs became spuriously associated with BMI among CRC cases, thus violating the MR independence assumption.

Simulation steps:

1. **Genotype simulation under HWE:**

- TNF-associated SNPs were generated as a function of their allele frequencies. For individual i at SNP j, genotype values were simulated as $g_{ij} \sim$ Binom(2,p_j) where p_j is the allele frequency of SNP j. This setup follows Hardy-Weinberg Equilibrium (HWE) (In the absence of selection, mutation, genetic drift, or other forces, allele frequencies p and q are constant between generations, so equilibrium is reached.), which assumes random mating and no evolutionary forces like selection or migration.
- The TNF values are a function of the TNF-α SNPs, such that $x_i = \sum g_{ij}\beta_j + e_j$ where $e_j \sim N(0, V_E)$. This is a classic polygenic model, where complex traits like TNF arise from many small genetic contributions plus non-genetic factors.
- The genetic variance (variation in TNF-α levels that can be attributed to inherited genetic differences) is $V_G = \sum 2p_j(1 - p_j)\beta_j^2$ and the residual variance is $V_E = V_P - V_G$. The phenotypic variance V_P is the variance of the observed, circulating level of TNF-α, as measured in the blood and, that was used to obtain the effect sizes.
- The phenotypic variance was indirectly derived from the reported heritability of TNF-α in the UK Biobank GWAS by Sun et al (2023). The total SNP-based heritability of TNF-α was estimated at $h^2 = 0.1167$, with the cis-pQTL component explaining 0.0114 of the variance. Because heritability is defined as the proportion of phenotypic variance explained by the genetic variance $H^2 = \frac{V_G}{V_P}$ we can use this equation to find the phenotypic variance.

2. **Confounder simulation:** BMI and age values were generated to approximate observed distributions at GECCO. Pre-diagnostic BMI distributions were based on Campbell et al. (2021), using data from GECCO and related cohorts. Age at baseline was simulated from the distribution reported by Wei et al. (2017) in the Nurses' Health Study (NHS). NHS is also part of the studies that we used to simulate BMI data.

3. **CRC incidence:** CRC incidence was simulated using a Cox proportional hazards model as a function of genetically predicted TNF-α, BMI, and age with censoring at 10 years. Effect estimates were taken from Campbell et al. (2021) for BMI, Wei et al. (2017) for age and Yuan et al. (2020) for TNF-α.

4. **CRC mortality:** CRC mortality status was simulated using an exponential proportional hazards model as a function of BMI and age, with censoring at 5 years. Effect estimates for BMI were extracted from the review by Kohls et al. (2022), and effect estimates for age (by age group) were taken from van Eeghen et al. (2015) We also simulated the death rate to match the one we have from GECCO.

5. **Case restriction:** Only CRC cases were retained for subsequent analyses.

6. **MR analysis:** SNP–exposure and SNP–outcome associations were estimated, and MR analyses performed to test whether TNF-α appeared associated with CRC-specific mortality, and whether TNF-α SNPs appeared associated with BMI among CRC cases.

7. **Repetition:** Steps 1–6 were repeated 1,000 times to obtain the distribution of MR estimates expected under collider bias alone.

Additional Table 6: Performance of Mendelian Randomization (MR) estimation of TNF-α on CRC-specific mortality across 1,000 Simulations

Metric	Estimate	MCSE
Bias	0.02	0.004
Empirical SE	0.13	0.003
Coverage (95%)	0.94	0.008
Type I error (p<0.05)	0.06	0.008

[a]Estimates are based on 1,000 simulated datasets under the null hypothesis of no causal effect of TNF-α on CRC-specific mortality.

[b]Bias is defined as the mean difference between the estimated and true log(HR).

[c]Empirical SE is the standard deviation of the simulated log(HR) estimates.

[d]Coverage is the proportion of 95% confidence intervals that contained the true null effect.

[e]Type I error is the proportion of simulations where $p < 0.05$.

[f]MCSE = Monte Carlo standard error, quantifying simulation uncertainty of each performance metric.
