# Supplementary material for: Associations of genetically predicted interleukin-6 and tumor necrosis factor signaling pathways with mortality among persons with colorectal cancer: a two-sample Mendelian randomization
Source: BMC Med. 2026 Mar 6;24:211. doi: 10.1186/s12916-026-04736-9 (PMC13063702; doi:10.1186/s12916-026-04736-9)
Supplement: Supplementary file 4 — Additional file 4: STROBE-MR checklist. Checklist of recommended items to address in reports of Mendelian randomization studies. [file 12916_2026_4736_MOESM4_ESM.pdf]

Additional file 4

STROBE-MR checklist of recommended items to address in reports of Mendelian randomization studies

Title: Associations of genetically predicted interleukin-6 and tumor necrosis factor signaling pathways with mortality among persons with colorectal cancer: A two-sample Mendelian Randomization

Item No.	Section	Checklist item	Page No.	Relevant text from manuscript
1	**TITLE and ABSTRACT**	Indicate Mendelian randomization (MR) as the study's design in the title and/or the abstract if that is a main purpose of the study	1	Title: Associations of genetically predicted interleukin-6 and tumor necrosis factor signaling pathways with mortality among persons with colorectal cancer: A two-sample Mendelian Randomization
	**INTRODUCTION**			
2	**Background**	Explain the scientific background and rationale for the reported study. What is the exposure? Is a potential causal relationship between exposure and outcome plausible? Justify why MR is a helpful method to address the study question	3,4	These cytokines play a crucial role in the inflammatory response, including hepatic production of acute-phase proteins such as C-reactive protein (CRP) (24). Several observational studies have reported positive associations between post-diagnostic and preoperative CRP concentrations and CRC prognosis (29, 30). In contrast, results from Mendelian Randomization (MR) studies have been inconsistent, with most studies showing no overall association of genetically predicted CRP concentrations on mortality in persons with CRC, although some found significant associations for individual SNPs (31-33).<br><br>While CRP has been widely studied, with no evidence for a causal role in CRC-specific mortality, here we investigated the inflammatory signatures of IL-6 and TNF-α in relation to mortality among individuals with CRC. Observational studies have reported elevated IL-6 and TNF-α levels in individuals with CRC compared to healthy controls (34-36), with higher IL-6 levels associated with more advanced disease, metastasis, and poorer survival (36-39). Some studies have also linked higher TNF-α levels to shorter overall survival, although

				findings are mixed (34, 40, 41). For the soluble receptors or IL6ST, evidence is limited. Understanding the potential roles of these cytokines is important, as it may provide insights into biological mechanisms underlying CRC progression and identify potential targets for improving prognosis and personalized treatment. Therefore, we explored whether the inflammatory signature defined by IL-6, TNF-α is associated with CRC-specific mortality in people with CRC using an MR approach.
3	**Objectives**	State specific objectives clearly, including pre-specified causal hypotheses (if any). State that MR is a method that, under specific assumptions, intends to estimate causal effects	4	We conducted a two-sample MR analysis to evaluate the potential relationship between genetically predicted levels of IL-6, sIL6-RA, IL6ST, TNF-α, sTNF-R1, and sTNF-R2 and CRC-specific mortality. MR is a technique that uses genetic variation as a tool to investigate genetic causal relationships between modifiable exposures and health outcomes (42). It is based on the principle that genetic variants are randomly assigned at conception, which makes them less susceptible to confounding factors that often affect observational studies (43). MR also helps to mitigate the issue of reverse causation, where disease progression may influence the exposure of interest — a particular concern in studies of survival (42)
	**METHODS**			
4	**Study design and data sources**	Present key elements of the study design early in the article. Consider including a table listing sources of data for all phases of the study. For each data source contributing to the analysis, describe the following:		
	a)	Setting: Describe the study design and the underlying population, if possible. Describe the setting, locations, and relevant dates, including periods of recruitment, exposure, follow-up, and data collection, when available.	4,5,6	Summary statistics for genetic associations with cytokine levels (exposures) and CRC-specific mortality (outcome) were obtained from separate, non-overlapping genome-wide association study (GWAS) datasets.<br>Exposure Data<br>Genetic instruments for circulating cytokines and soluble receptors were derived separately from two recent large protein quantitative trait loci (pQTL) GWAS. The first by Ferkingstad et al, was conducted in 35,559 Icelanders

within the deCODE study, where plasma levels of 4,719 proteins were measured using the SomaScan v4 platform and tested against 27.2 million sequence variants imputed from whole-genome sequencing of 49,708 individuals (46). Association analyses were adjusted for age, sex, and sample age (time since blood collection). The second by Sun et al, was based on 46,861 participants in the UK Biobank (UKBB), with plasma protein levels measured using the Olink Explore 1536 platform and analyzed against genome-wide genetic variation (47). Association analyses adjusted for age, age2, sex, sex×age, age2×sex, batch, UKBB centre, UKBB genetic array, time between blood sampling and measurement and the first 20 genetic principal components. Both GWAS were included to make use of independent large-scale datasets and ensure that the most well-powered instruments could be selected for each biomarker, and to assess whether results were consistent across studies.

From both GWAS, we extracted summary statistics for cis-acting variants (±500 kb of the gene encoding each biomarker). We first considered variants reaching conventional genome-wide significance ($p < 5 \times 10^{-8}$). No suitable cis SNPs were identified for IL-6 in either GWAS. For sIL6-RA, the resulting total R2 of SNPs was sufficient to apply conventional LD clumping at $r^2 < 0.001$. For other biomarkers, the LD threshold was relaxed to $r^2 < 0.1$ to increase instrument numbers and improve statistical power. Instrument sets with total variance explained $R^2 < 1\%$ were excluded. We also explored a more lenient threshold ($p < 1 \times 10^{-6}$), but this did not identify variants for IL-6 either and only modestly increased instrument numbers for other biomarkers, without substantially improving power.

Outcome data

Genetic association estimates (EAF, beta coefficients, SEs, p-values, and sample sizes) for colorectal cancer (CRC)-specific mortality were obtained from the Genetics and Epidemiology of Colorectal Cancer Consortium (GECCO) and the Colon Cancer Family Registry (CCFR) (48). These consortia had combined

				data from 15 cohort and clinical studies, including 16,964 individuals of European ancestry diagnosed with incident, invasive CRC. During a median follow-up of 13.8 years, 4,010 CRC-related deaths had been recorded. Genotyping had been conducted across multiple platforms, followed by standard quality control procedures, and imputation had been performed using the Haplotype Reference Consortium reference panel (release 1.1). All analyses were performed by the consortia prior to data provision. Specifically, Cox proportional hazards regression models were used to assess associations between individual SNPs and CRC-specific mortality under an additive genetic model. Models were adjusted for age at diagnosis, sex, study-specific factors, genotyping platform, and the first five principal components to account for population structure. In addition to overall analyses, stratified analyses were conducted by anatomical tumor site: proximal colon (6,214 cases, 1,433 deaths), distal colon (4,881 cases, 978 deaths), rectum (4,749 cases, 1,045 deaths) and by tumor stage: stage I (3,338 cases, 157 deaths), stages II–III (6,420 cases, 1,209 deaths), stage IV (1,847 cases, 1,448 deaths). Further details on cohort characteristics, genotyping, quality control, and imputation procedures are available in the original GWAS publication (49).
	b)	Participants: Give the eligibility criteria, and the sources and methods of selection of participants. Report the sample size, and whether any power or sample size calculations were carried out prior to the main analysis	5,7,8	The first by Ferkingstad et al, was conducted in 35,559 Icelanders within the deCODE study, where plasma levels of 4,719 proteins were measured using the SomaScan v4 platform and tested against 27.2 million sequence variants imputed from whole-genome sequencing of 49,708 individuals (46). Association analyses were adjusted for age, sex, and sample age (time since blood collection). The second by Sun et al, was based on 46,861 participants in the UK Biobank (UKBB), with plasma protein levels measured using the Olink Explore 1536 platform and analyzed against genome-wide genetic variation (47). Association analyses adjusted for age, age2, sex, sex×age, age2×sex, batch,

			UKBB centre, UKBB genetic array, time between blood sampling and measurement and the first 20 genetic principal components. Both GWAS were included to make use of independent large-scale datasets and ensure that the most well-powered instruments could be selected for each biomarker, and to assess whether results were consistent across studies.<br>Genetic association estimates (EAF, beta coefficients, SEs, p-values, and sample sizes) for colorectal cancer (CRC)-specific mortality were obtained from the Genetics and Epidemiology of Colorectal Cancer Consortium (GECCO) and the Colon Cancer Family Registry (CCFR) (48). These consortia had combined data from 15 cohort and clinical studies, including 16,964 individuals of European ancestry diagnosed with incident, invasive CRC. During a median follow-up of 13.8 years, 4,010 CRC-related deaths had been recorded.<br>**Calculations of the minimally detectable effect (Hazard Ratio)**<br>We calculated the minimally detectable hazard ratio (HR) for CRC-specific mortality as a binary outcome using the method described by Burgess et al (45) with a type 1 error α=0.05. This method does not take the time until the end of follow-up into account. Based on the number of total CRC cases and deaths in the outcome dataset, our study had at least 80% power to detect the following HRs per SD: in deCODE, 1.05 for sIL6-RA, 1.14 for IL6ST, and 1.42 for both sTNF-R1 and sTNF-R2; in UK Biobank, 1.07 for sIL6-RA, 1.21 for IL6ST, 1.24 for TNF-α, and 1.23 for sTNF-R2 (Additional file 2).
c)	Describe measurement, quality control and selection of genetic variants	5	From both GWAS, we extracted summary statistics for cis-acting variants (±500 kb of the gene encoding each biomarker). We first considered variants reaching conventional genome-wide significance ($p < 5 \times 10^{-8}$). No suitable cis SNPs were identified for IL-6 in either GWAS. For sIL6-RA, the resulting total R2 of SNPs was sufficient to apply conventional LD clumping at $r^2 < 0.001$. For other biomarkers, the LD threshold was relaxed to $r^2 < 0.1$ to increase instrument numbers and improve statistical power. Instrument sets with total

			variance explained $R^2 < 1\%$ were excluded. We also explored a more lenient threshold ($p < 1 \times 10^{-6}$), but this did not identify variants for IL-6 either and only modestly increased instrument numbers for other biomarkers, without substantially improving power.<br><br>Following these criteria, the deCODE GWAS provided instruments for sIL6-RA (13 SNPs, $R^2 = 49\%$), IL6ST (23 SNPs, $R^2 = 6.9\%$), sTNF-R1 (4 SNPs, $R^2 = 1.0\%$), and sTNF-R2 (5 SNPs, $R^2 = 1.0\%$). The UKBB GWAS provided instruments for sIL6-RA (11 SNPs, $R^2 = 25\%$), IL6ST (12 SNPs, $R^2 = 3.3\%$), TNF-α (14 SNPs, $R^2 = 2.6\%$), and sTNF-R2 (11 SNPs, $R^2 = 2.8\%$). For all biomarkers the F-statistics were above 10. Characteristics of all SNP instruments, including effect estimates and strength metrics, are shown in Additional File 1, and the SNP selection workflow is illustrated in Figure 1.
d)	For each exposure, outcome, and other relevant variables, describe methods of assessment and diagnostic criteria for diseases	3,4	Colorectal cancer (CRC) is the third most common in terms of incidence and the second leading cause of cancer-related deaths worldwide (1). Significant progress has been made in understanding the risk factors for CRC development, such as diet, nutrition and lifestyle factors, including obesity (2-4). Thus, the most recent report of the World Cancer Research Fund (WCRF) concluded that there is strong evidence that being overweight or obese is associated with a higher CRC risk (5, 6), which is supported by large Mendelian Randomization (MR) studies (7-11). However, less is known about factors that influence prognosis and mortality in people with CRC.<br><br>Several cytokines, including interleukin-6 (IL-6) and tumor necrosis factor-alpha (TNF-α), are upregulated in CRC and are associated with poor prognosis in patients (20). Both are produced by a variety of cells including adipose tissue–resident macrophages as well as cells in other tissues, and their blood concentrations are positively correlated with adiposity measures (21-24).<br><br>In addition, the soluble IL-6 receptor (sIL6-RA) circulates in blood, serving as binding reservoir but also modulating IL-6–mediated signaling. sIL6-RA also enables trans-signaling, allowing IL-6 to act on cells that

				do not express the membrane-bound receptor, thereby broadening its pro-inflammatory effects. Thus, when IL-6 binds to either the membrane-bound IL6-RA or to sIL6-RA, the complex may associate with the signal transducer gp130 (IL6ST), thereby initiating downstream activation of the JAK/STAT pathway and promoting inflammatory responses (25, 26). Soluble receptors of TNF-α (sTNF-R1, sTNF-R2) primarily function to bind and regulate circulating TNF-α, acting as reservoirs that can either stabilize or attenuate TNF-α activity depending on the physiological context (27, 28). These cytokines play a crucial role in the inflammatory response, including hepatic production of acute-phase proteins such as C-reactive protein (CRP) (24). Several observational studies have reported positive associations between post-diagnostic and preoperative CRP concentrations and CRC prognosis (29, 30). In contrast, results from Mendelian Randomization (MR) studies have been inconsistent, with most studies showing no overall association of genetically predicted CRP concentrations on mortality in persons with CRC, although some found significant associations for individual SNPs (31-33).
		e) Provide details of ethics committee approval and participant informed consent, if relevant	Declarations	The GWAS summary statistics used for the exposures were publicly available and did not require additional ethical approval. For the GECCO data used for CRC-specific mortality outcomes, each participating study obtained ethical approval from their respective institutional review boards, and all participants provided informed consent.
5	**Assumptions**	Explicitly state the three core IV assumptions for the main analysis (relevance, independence and exclusion restriction) as well assumptions for any additional or sensitivity analysis	4	In all MR analysis, the selected genetic instruments have to meet the following criteria: (a) be strongly associated with the circulating levels of the cytokine (relevance assumption), (b) be independent of any potential confounder of the cytokine-CRC mortality association (independence assumption) and (c) affect CRC mortality only through the cytokine being instrumented and not via any other biological pathway (exclusion restriction assumption)

6	**Statistical methods: main analysis**	Describe statistical methods and statistics used	7	**Statistical Analysis**<br>**Mendelian Randomization Analysis**<br>Two-sample MR using summary association data was performed for CRC-specific mortality overall, as well as stratified by anatomical tumor subsite and stage of cancer. MR estimates were obtained using the random-effects inverse variance weighted (IVW) method. To account for multiple testing across the four exposures available in each GWAS, we applied Bonferroni correction (corrected α = 0.0125).
	a)	Describe how quantitative variables were handled in the analyses (i.e., scale, units, model)	7	associations are reported per one standard deviation (SD) increase in biomarker levels
	b)	Describe how genetic variants were handled in the analyses and, if applicable, how their weights were selected	7	For inflammatory biomarkers with instruments available from both GWAS (deCODE and UK Biobank), analyses were conducted separately for each dataset. To account for dependency among SNPs, a correlation matrix of the genetic instruments was incorporated for all biomarkers except sIL6-RA.
	c)	Describe the MR estimator (e.g. two-stage least squares, Wald ratio) and related statistics. Detail the included covariates and, in case of two-sample MR, whether the same covariate set was used for adjustment in the two samples	5	Genetic instruments for circulating cytokines and soluble receptors were derived separately from two recent large protein quantitative trait loci (pQTL) GWAS. The first by Ferkingstad et al, was conducted in 35,559 Icelanders within the deCODE study, where plasma levels of 4,719 proteins were measured using the SomaScan v4 platform and tested against 27.2 million sequence variants imputed from whole-genome sequencing of 49,708 individuals (46). Association analyses were adjusted for age, sex, and sample age (time since blood collection). The second by Sun et al, was based on 46,861 participants in the UK Biobank (UKBB), with plasma protein levels measured using the Olink Explore 1536 platform and analyzed against genome-wide genetic variation (47). Association analyses adjusted for age, age2, sex, sex×age, age2×sex, batch, UKBB centre, UKBB genetic array, time between blood sampling and measurement and the first 20 genetic principal components. MR estimates were obtained using

				the random-effects inverse variance weighted (IVW) method.
		d) Explain how missing data were addressed		Not Applicable
		e) If applicable, indicate how multiple testing was addressed	7	To account for multiple testing across the four exposures available in each GWAS, we applied Bonferroni correction (corrected α = 0.0125).
7	**Assessment of assumptions**	Describe any methods or prior knowledge used to assess the assumptions or justify their validity		
8	**Sensitivity analyses and additional analyses**	Describe any sensitivity analyses or additional analyses performed (e.g. comparison of effect estimates from different approaches, independent replication, bias analytic techniques, validation of instruments, simulations)	7	**Sensitivity analyses**<br>To examine potential violation of the second and third MR assumptions the Cohran's Q statistic was calculated which assesses the degree to which variation in the individual SNP-specific effect estimates reflects true heterogeneity between genetic variants rather than random variation due to sampling error (50). MR-Egger regression was performed to detect potential directional pleiotropy, with statistical evidence assessed by the intercept term being different from zero. In addition, leave-one-out (LOO) analyses were conducted by iteratively excluding each genetic variant to assess the influence of individual SNPs on the overall estimate. When a correlation matrix was included to account for linkage disequilibrium between genetic instruments, statistical sensitivity methods such as weighted median and MR-PRESSO (which detects and corrects for potential outlier variants that may bias the associations) could not be performed, as these methods require independent instruments. Therefore, they were implemented only for sIL6-RA. Finally, we performed colocalization analyses, where relevant, to assess whether genetic variants associated with inflammatory protein levels also influenced CRC-specific mortality through shared causal variants.
9	**Software and pre-registration**			

		a) Name statistical software and package(s), including version and settings used	7	Analyses were implemented in the statistical software R (version 4.4.3) using the TwoSampleMR, MendelianRandomization, MRPRESO and coloc packages.
		b) State whether the study protocol and details were pre-registered (as well as when and where)		Not Applicable
	**RESULTS**			
10	**Descriptive data**			
		a) Report the numbers of individuals at each stage of included studies and reasons for exclusion. Consider use of a flow diagram	5	and soluble receptors were derived separately from two recent large protein quantitative trait loci (pQTL) GWAS. The first by Ferkingstad et al, was conducted in 35,559 Icelanders within the deCODE study, where plasma levels of 4,719 proteins were measured using the SomaScan v4 platform and tested against 27.2 million sequence variants imputed from whole-genome sequencing of 49,708 individuals (46). Association analyses were adjusted for age, sex, and sample age (time since blood collection). The second by Sun et al, was based on 46,861 participants in the UK Biobank (UKBB), with plasma protein levels measured using the Olink Explore 1536 platform and analyzed against genome-wide genetic variation (47). Association analyses adjusted for age, age2, sex, sex×age, age2×sex, batch, UKBB centre, UKBB genetic array, time between blood sampling and measurement and the first 20 genetic principal components. Both GWAS were included to make use of independent large-scale datasets and ensure that the most well-powered instruments could be selected for each biomarker, and to assess whether results were consistent across studies.
		b) Report summary statistics for phenotypic exposure(s), outcome(s), and other relevant variables (e.g. means, SDs, proportions)	5,7	After harmonization with the outcome dataset, all selected SNPs were retained for sIL6-RA and sTNF-R2 from the deCODE GWAS, while 19 SNPs remained for IL6ST and 3 SNPs for sTNF-R1. For the UKBB GWAS, all SNPs were retained for sIL6-RA, and 9 SNPs each for IL6ST and TNF-α, as well as 10 SNPs for sTNF-R2 Genetic association estimates (EAF, beta coefficients, SEs, p-values, and sample sizes) for colorectal cancer

				(CRC)-specific mortality were obtained from the Genetics and Epidemiology of Colorectal Cancer Consortium (GECCO) and the Colon Cancer Family Registry (CCFR) (48). These consortia had combined data from 15 cohort and clinical studies, including 16,964 individuals of European ancestry diagnosed with incident, invasive CRC. During a median follow-up of 13.8 years, 4,010 CRC-related deaths had been recorded. Genotyping had been conducted across multiple platforms, followed by standard quality control procedures, and imputation had been performed using the Haplotype Reference Consortium reference panel (release 1.1). All analyses were performed by the consortia prior to data provision. Specifically, Cox proportional hazards regression models were used to assess associations between individual SNPs and CRC-specific mortality under an additive genetic model. Models were adjusted for age at diagnosis, sex, study-specific factors, genotyping platform, and the first five principal components to account for population structure. In addition to overall analyses, stratified analyses were conducted by anatomical tumor site: proximal colon (6,214 cases, 1,433 deaths), distal colon (4,881 cases, 978 deaths), rectum (4,749 cases, 1,045 deaths) and by tumor stage: stage I (3,338 cases, 157 deaths), stages II–III (6,420 cases, 1,209 deaths), stage IV (1,847 cases, 1,448 deaths). Further details on cohort characteristics, genotyping, quality control, and imputation procedures are available in the original GWAS publication (49).
		c) If the data sources include meta-analyses of previous studies, provide the assessments of heterogeneity across these studies		-
		d) For two-sample MR:<br>i. Provide justification of the similarity of the genetic variant-exposure associations between the exposure and outcome samples<br>ii. Provide information on the number of individuals who overlap between the exposure and outcome studies	4	Summary statistics for genetic associations with cytokine levels (exposures) and CRC-specific mortality (outcome) were obtained from separate, non-overlapping genome-wide association study (GWAS) datasets
11	**Main results**			

a)	Report the associations between genetic variant and exposure, and between genetic variant and outcome, preferably on an interpretable scale	5,7	Characteristics of all SNP instruments, including effect estimates and strength metrics, are shown in Additional File 1. CRC mortality estimates for each selected biomarker-associated SNP are presented in Additional file 1.
b)	Report MR estimates of the relationship between exposure and outcome, and the measures of uncertainty from the MR analysis, on an interpretable scale, such as odds ratio or relative risk per SD difference	10,11	For sIL6-RA, the IVW analysis indicated higher CRC-specific mortality in both GWAS (HR per 1 SD increase: 1.06; 95% CI: 1.00–1.12 using deCODE-SNPs and HR: 1.09; 95% CI: 1.02–1.17 using UKBB-SNPs) (Table 1). The association using UKBB-SNPs remained significant (p-value: raw = 0.012, adjusted = 0.047) after Bonferroni correction, whereas the one based on deCODE-SNPs (p-value: raw = 0.042, adjusted = 0.167) did not. Subsite- and stage-specific analyses were broadly consistent across GWAS, with no further associations observed.<br><br>For IL6ST, the IVW analysis suggested no association with CRC-specific mortality overall (HR: 1.04; 95% CI: 0.90–1.21 using deCODE-SNPs and HR: 1.11; 95% CI: 0.87–1.42 using UKBB-SNPs) (Table 1). In stage-stratified analyses, positive associations were observed for Stages 2/3 disease (HR: 1.45; 95% CI: 1.10–1.91; p-value: raw = 0.009, adjusted = 0.036 using deCODE-SNPs and HR: 1.87; 95% CI: 1.22–2.89; p-value: raw = 0.004, adjusted = 0.018 using UKBB-SNPs) and both remained significant after Bonferroni correction. Subsite-specific analyses did not indicate other associations.<br><br>For genetically predicted TNF-α, the IVW analysis using UKBB-SNPs suggested no association with CRC-specific mortality (HR: 1.19; 95% CI: 0.79–1.77). Similarly, for the soluble receptors, sTNF-R1 (HR: 0.98; 95% CI: 0.70–1.36 using deCODE SNPs) and sTNF-R2 (HR: 0.95; 95% CI: 0.67–1.33 using deCODE-SNPS and HR: 1.02; 95% CI: 0.63–1.63 using UKBB-SNPs) no associations were observed with overall CRC-specific mortality. Subsite- and stage-stratified analyses did not reveal any further associations (Table 1).
c)	If relevant, consider translating estimates of relative risk into absolute risk for a meaningful time period		Not Applicable

		d)	Consider plots to visualize results (e.g. forest plot, scatterplot of associations between genetic variants and outcome versus between genetic variants and exposure)		Additional file 3
12	**Assessment of assumptions**				
		a)	Report the assessment of the validity of the assumptions	5,11,12	Following these criteria, the deCODE GWAS provided instruments for sIL6-RA (13 SNPs, $R^2 = 49\%$), IL6ST (23 SNPs, $R^2 = 6.9\%$), sTNF-R1 (4 SNPs, $R^2 = 1.0\%$), and sTNF-R2 (5 SNPs, $R^2 = 1.0\%$). The UKBB GWAS provided instruments for sIL6-RA (11 SNPs, $R^2 = 25\%$), IL6ST (12 SNPs, $R^2 = 3.3\%$), TNF-α (14 SNPs, $R^2 = 2.6\%$), and sTNF-R2 (11 SNPs, $R^2 = 2.8\%$). For all biomarkers the F-statistics were above 10. Characteristics of all SNP instruments, including effect estimates and strength metrics, are shown in Additional File 1, and the SNP selection workflow is illustrated in Figure 1.<br><br>Across both biomarkers, MR-Egger regression provided effect estimates of similar direction and magnitude, (Table 1). Weighted median analyses for sIL6-RA produced results consistent with the IVW estimates, and MR-PRESSO did not identify outlier SNPs (Additional file 2). No evidence of substantial heterogeneity was indicated with non-significant Cochran's Q statistics (all $p > 0.05$). Evidence for horizontal pleiotropy was observed only in the IL6ST distal colon analysis using deCODE-SNPs and Stage 4 disease using UKBB-SNPs (MR-Egger intercept $p = 0.04$), though these p-values were marginal and since MR-Egger estimates were highly consistent with the IVW results, pleiotropy is unlikely to materially affect the conclusions. LOO analyses for sIL6-RA and IL6ST indicated that results were generally robust. Minor shifts in effect estimates were observed when excluding individual SNPs in some subsite-specific analyses, but overall patterns remained consistent using either deCODE or UKBB SNPs (Additional file 3).<br><br>Given the observed associations for sIL6-RA and IL6ST, we conducted colocalization analyses in the overall CRC sample to examine whether genetic variants at these loci shared causal signals with CRC-specific mortality.

					Analyses were performed separately using instruments from the deCODE and UKBB GWAS, and results were highly consistent across datasets.<br><br>For TNF-α and its soluble receptors, MR-Egger regression provided effect estimates consistent with the IVW results, though with wider confidence intervals (Table 1). No evidence of substantial heterogeneity was indicated, except in analyses of TNF-α for distal colon cancer and sTNF-R2 for distal colon cancer using deCODE SNPs (Cochran's p =0.04 for both) (Additional file 2). A weak indication of pleiotropy was observed only for sTNF-R2 in the distal colon analysis using deCODE-SNPs (MR-Egger intercept p = 0.04), though the MR-Egger estimates were consistent with the IVW results. LOO analyses did not suggest that results were driven by any single SNP for TNF-α, sTNF-R1, or sTNF-R2.
		b)	Report any additional statistics (e.g., assessments of heterogeneity across genetic variants, such as I^2, Q statistic or E-value)	11	Heterogeneity and pleiotropy tests showed no evidence of violation of MR assumptions, with non-significant Cochran's Q statistics (all p > 0.05) and no evidence of directional pleiotropy.
13	**Sensitivity analyses and additional analyses**				
		a)	Report any sensitivity analyses to assess the robustness of the main results to violations of the assumptions8	11,12	Across both biomarkers, MR-Egger regression provided effect estimates of similar direction and magnitude, (Table 1). Weighted median analyses for sIL6-RA produced results consistent with the IVW estimates, and MR-PRESSO did not identify outlier SNPs (Additional file 2). No evidence of substantial heterogeneity was indicated with non-significant Cochran's Q statistics (all p > 0.05). Evidence for horizontal pleiotropy was observed only in the IL6ST distal colon analysis using deCODE-SNPs and Stage 4 disease using UKBB-SNPs (MR-Egger intercept p = 0.04), though these p-values were marginal and since MR-Egger estimates were highly consistent with the IVW results, pleiotropy is unlikely to materially affect the conclusions. LOO analyses for sIL6-RA and IL6ST indicated that results

			were generally robust. Minor shifts in effect estimates were observed when excluding individual SNPs in some subsite-specific analyses, but overall patterns remained consistent using either deCODE or UKBB SNPs (Additional file 3).<br><br>Given the observed associations for sIL6-RA and IL6ST, we conducted colocalization analyses in the overall CRC sample to examine whether genetic variants at these loci shared causal signals with CRC-specific mortality.<br><br>For TNF-α and its soluble receptors, MR-Egger regression provided effect estimates consistent with the IVW results, though with wider confidence intervals (Table 1). No evidence of substantial heterogeneity was indicated, except in analyses of TNF-α for distal colon cancer and sTNF-R2 for distal colon cancer using deCODE SNPs (Cochran's p =0.04 for both) (Additional file 2). A weak indication of pleiotropy was observed only for sTNF-R2 in the distal colon analysis using deCODE-SNPs (MR-Egger intercept p = 0.04), though the MR-Egger estimates were consistent with the IVW results. LOO analyses did not suggest that results were driven by any single SNP for TNF-α, sTNF-R1, or sTNF-R2.
b)	Report results from other sensitivity analyses or additional analyses	13	**Collider Bias**<br><br>We performed simulations under the assumption of no effect of TNF-α on CRC-specific mortality. In this setting, any associations observed would necessarily arise from collider bias.<br><br>On average, the MR estimator produced a very small upward bias (mean β = 0.022, HR ≈ 1.02). Across 1,000 simulations, the distribution of effect estimates varied around the null, with hazard ratios ranging roughly between 0.81 and 1.31. This indicates that while individual simulated datasets can yield estimates away from the null, the average result remained close to no effect. The performance metrics confirmed this pattern: bias was small (0.022), type I error was slightly inflated (6.1% vs expected 5%), and coverage of the 95% confidence interval was slightly below nominal (93.9%).

			To directly assess collider bias, we examined associations between TNF-α SNPs and BMI among CRC cases. On average, 0.14 SNPs per run showed a spurious association, and in ~13% of simulations at least one SNP was significant (maximum observed per run = 2 SNPs). This indicates that collider bias was present but modest, and unlikely to explain large effect estimates. Taken together, the simulations suggest that in the absence of an effect of TNF-α on CRC-specific mortality, MR estimates remain close to no effect, with only small bias and occasional spurious SNP–BMI associations due to collider bias (Additional files 2 and 3).
c)	Report any assessment of direction of causal relationship (e.g., bidirectional MR)		-
d)	When relevant, report and compare with estimates from non-MR analyses	14,15	Evidence from observational studies suggests that elevated IL-6 levels may be associated with poorer survival in individuals with CRC. Several case-control studies have reported higher circulating post-diagnostic IL-6 levels in individuals with CRC compared to healthy controls (34-36). In addition, some of these same studies (35, 36), along with others (37, 38), have shown that higher IL-6 levels are related to more advanced disease, metastasis, and shorter survival in CRC patients. A systematic review and meta-analysis also confirmed that serum IL-6 expression was highly correlated with poor 5-year overall survival (39). Another study based on post-treatment plasma samples from CRC patients in the Seattle Colon Cancer Family Registry reported that individuals with stage II or III CRC in the highest category of IL-6 concentrations had a significantly higher risk of CRC-specific mortality (HR = 5.02, 95% CI: 2.92–8.59) compared to those with the lowest levels, with this association persisting over 10 years of follow-up (64). For the soluble IL-6 receptor the literature is more limited, with one study having found that high expression of sIL6-RA in the tumor epithelium was associated with reduced cancer-specific survival in patients with right-sided colon cancer (65).<br><br>Several observational studies have also reported elevated TNF-α levels in individuals with CRC compared to

				healthy controls (34, 37, 41). However, findings regarding its association with prognosis are mixed. Two studies reported significantly poorer overall survival in people with CRC who had higher TNF-α levels (34, 41). A third study by Olsen et al. (40) found that individuals in the highest tertile of TNF-α did not have significantly higher CRC-specific mortality in univariate analysis compared to those in the lowest tertile (HR = 1.5; 95% CI: 0.7–3.0), but the association became statistically significant after adjustment for clinical covariates (HR = 2.3; 95% CI: 1.0–5.4). For TNF receptors, literature on their prognostic value in CRC is scarce. However, in the study by Babic et al (66), higher pre-diagnostic sTNF-R2 levels were not associated with CRC-specific mortality (HR for highest vs lowest quartile=1.23; 95% CI, 0.72–2.08). These mixed findings, together with the lack of associations in our MR analyses, underline the complexity of TNF-α as a biomarker and highlight the need for further research to clarify its role in CRC prognosis.
		e) Consider additional plots to visualize results (e.g., leave-one-out analyses)		Additional file 3
	**DISCUSSION**			
14	**Key results**	Summarize key results with reference to study objectives	13	In this two-sample MR study, we investigated the role of genetically predicted IL-6 signaling (proxied by sIL6-RA and IL6ST), TNF-α, and soluble TNF receptors (sTNF-R1 and sTNF-R2) in colorectal cancer (CRC)-specific mortality among individuals with CRC. We found modest evidence for IL-6 signaling in relation to CRC-specific mortality. Genetically predicted higher sIL6-RA levels were consistently associated with slightly increased mortality across both GWAS, with the association using UKBB-SNPs surviving the Bonferroni correction. IL6ST showed associations only in stage-stratified analyses, with higher genetically predicted levels linked to increased mortality in stage 2/3 disease. In contrast, we found no consistent evidence that TNF-α or its soluble receptors (sTNF-R1 and sTNF-R2) were associated with CRC-specific mortality. Sensitivity analyses, including MR-Egger, weighted median, MR-

				PRESSO, heterogeneity and pleiotropy testing, and LOO analyses, indicated that the results were generally robust, with no evidence of directional pleiotropy and no indication that findings were materially driven by single SNPs. Collider bias simulations further suggested that any bias introduced by restricting analyses to CRC cases was modest and unlikely to generate spurious associations. Overall, our findings provide some evidence for a role of IL-6 signaling in CRC-specific mortality, although of limited magnitude, while not supporting a major contribution of TNF-α pathways.
15	**Limitations**	Discuss limitations of the study, taking into account the validity of the IV assumptions, other sources of potential bias, and imprecision. Discuss both direction and magnitude of any potential bias and any efforts to address them	15	The main limitation of our study is the limited number of SNPs available for TNF-α and its soluble receptors. This resulted in low statistical power, and sometimes wide confidence intervals, and restricted our ability to perform formal collider bias adjustments, which require a large set of well-powered instruments. To address this, we implemented a simulation approach; however, as highlighted by Mitchell et al (53)., simulated data may not fully reflect reality and can be misleading, with no formal recommendations on how to directly compare simulated with observed effects. Another potential limitation is that the genetic associations with cytokine levels used as instruments were derived from general population cohorts, whereas our outcome was CRC-specific mortality among diagnosed patients. It is possible that disease status or treatment could alter cytokine regulation, meaning that the SNP–cytokine effects in patients with CRC may differ from those in the general population, potentially introducing bias. More generally, as with all MR studies, uncertainties remain regarding potential violations of the underlying assumptions, such as unrecognized pleiotropy or weak instrument bias, which cannot be fully excluded despite extensive sensitivity analyses. Finally, the generalizability of our findings is currently limited to populations of European ancestry, and to genetic proxies of chronic cytokine exposure rather than short-term, treatment-related fluctuations. Future studies in more diverse populations and with time-specific biomarker

				measurements are warranted to fully characterize these associations
16	**Interpretation**			
		a) Meaning: Give a cautious overall interpretation of results in the context of their limitations and in comparison with other studies	14,15	Overall, our findings provide some evidence for a role of IL-6 signaling in CRC-specific mortality, although of limited magnitude, while not supporting a major contribution of TNF-α pathways.<br><br>Evidence from observational studies suggests that elevated IL-6 levels may be associated with poorer survival in individuals with CRC. Several case-control studies have reported higher circulating post-diagnostic IL-6 levels in individuals with CRC compared to healthy controls (34-36). In addition, some of these same studies (35, 36), along with others (37, 38), have shown that higher IL-6 levels are related to more advanced disease, metastasis, and shorter survival in CRC patients. A systematic review and meta-analysis also confirmed that serum IL-6 expression was highly correlated with poor 5-year overall survival (39). Another study based on post-treatment plasma samples from CRC patients in the Seattle Colon Cancer Family Registry reported that individuals with stage II or III CRC in the highest category of IL-6 concentrations had a significantly higher risk of CRC-specific mortality (HR = 5.02, 95% CI: 2.92–8.59) compared to those with the lowest levels, with this association persisting over 10 years of follow-up (64). For the soluble IL-6 receptor the literature is more limited, with one study having found that high expression of sIL6-RA in the tumor epithelium was associated with reduced cancer-specific survival in patients with right-sided colon cancer (65). However, these observational findings are based on cytokine concentrations measured after cancer diagnosis and treatment, which are likely influenced by factors such as tumor burden, systemic inflammation, and recent chemotherapy or radiotherapy. These post-treatment levels may not reflect pre-diagnostic or causal cytokine activity. In addition, most observational studies assess overall survival, which includes deaths from causes unrelated to CRC, limiting comparability with our outcome of CRC-specific

			mortality. We observed only modest associations of IL-6 signaling with CRC-specific mortality. Our findings align with the recent MR study from the GECCO consortium, which found no association between genetically predicted C-reactive protein (CRP) levels and CRC-specific survival (31). As CRP is a downstream acute-phase protein primarily stimulated by IL-6, the lack of association with CRP suggests that systemic inflammatory responses may not affect CRC-specific prognosis, although our results indicate that components of the IL-6 signaling pathway could still play a role. Several observational studies have also reported elevated TNF-α levels in individuals with CRC compared to healthy controls (34, 37, 41). However, findings regarding its association with prognosis are mixed. Two studies reported significantly poorer overall survival in people with CRC who had higher TNF-α levels (34, 41). A third study by Olsen et al. (40) found that individuals in the highest tertile of TNF-α did not have significantly higher CRC-specific mortality in univariate analysis compared to those in the lowest tertile (HR = 1.5; 95% CI: 0.7–3.0), but the association became statistically significant after adjustment for clinical covariates (HR = 2.3; 95% CI: 1.0–5.4). For TNF receptors, literature on their prognostic value in CRC is scarce. However, in the study by Babic et al (66), higher pre-diagnostic sTNF-R2 levels were not associated with CRC-specific mortality (HR for highest vs lowest quartile=1.23; 95% CI, 0.72–2.08). These mixed findings, together with the lack of associations in our MR analyses, underline the complexity of TNF-α as a biomarker and highlight the need for further research to clarify its role in CRC prognosis.
b)	Mechanism: Discuss underlying biological mechanisms that could drive a potential causal relationship between the investigated exposure and the outcome, and whether the gene-environment equivalence assumption is reasonable. Use causal language carefully, clarifying that IV estimates may provide causal effects only under certain assumptions	15	The potential biological mechanisms linking IL-6 and TNF-α to CRC mortality include chronic inflammation, immune modulation (67), and promotion of tumor growth and metastasis through pathways such as cell proliferation (68) and angiogenesis (69). Although we found little evidence that these genetically predicted cytokine levels influence CRC-specific mortality, the

				modest association observed for IL-6 signaling via sIL6-RA suggests that this pathway may still play a role.
		c) Clinical relevance: Discuss whether the results have clinical or public policy relevance, and to what extent they inform effect sizes of possible interventions	15	Clinically, IL-6 and TNF-α may still be useful as indicators of disease activity or inflammation, rather than as direct causes of poor outcomes. Such use would relate to cytokine levels measured after diagnosis, and further studies using observed biomarker data are needed to determine their relevance in this context.
17	**Generalizability**	Discuss the generalizability of the study results (a) to other populations, (b) across other exposure periods/timings, and (c) across other levels of exposure	15,16	Another potential limitation is that the genetic associations with cytokine levels used as instruments were derived from general population cohorts, whereas our outcome was CRC-specific mortality among diagnosed patients. It is possible that disease status or treatment could alter cytokine regulation, meaning that the SNP–cytokine effects in patients with CRC may differ from those in the general population, potentially introducing bias. More generally, as with all MR studies, uncertainties remain regarding potential violations of the underlying assumptions, such as unrecognized pleiotropy or weak instrument bias, which cannot be fully excluded despite extensive sensitivity analyses. Finally, the generalizability of our findings is currently limited to populations of European ancestry, and to genetic proxies of chronic cytokine exposure rather than short-term, treatment-related fluctuations.
	**OTHER INFORMATION**			
18	**Funding**	Describe sources of funding and the role of funders in the present study and, if applicable, sources of funding for the databases and original study or studies on which the present study is based	Declarations	This study did not receive specific project funding and was supported through intramural funding.<br>GECCO consortium funding: Additional file 5
19	**Data and data sharing**	Provide the data used to perform all analyses or report where and how the data can be accessed, and reference these sources in the article. Provide the statistical code needed to reproduce the results in the article, or report whether the code is publicly accessible and if so, where	Declarations	The summary statistics used in this study for all MR analyses are provided in Additional file 1 for replication purposes. The summary-level GWAS data on outcomes used in this study are available following an application to the Genetics and Epidemiology of Colorectal Cancer Consortium (GECCO).

20	**Conflicts of Interest**	All authors should declare all potential conflicts of interest	Declarations	The authors declare no conflicts of interest.

This checklist is copyrighted by the Equator Network under the Creative Commons Attribution 3.0 Unported (CC BY 3.0) license.
